# Picalm coordinates clathrin-mediated endocytosis and actin remodeling during myogenesis

**DOI:** 10.1016/j.molmet.2026.102351

**Published:** 2026-03-13

**Authors:** Jasmin Gaugel, Neele Haacke, Benno Kuropka, Markus Jähnert, Julia Rominger, Wenke Jonas, Thilo Speckmann, Niclas Rausch, Maximilian Kleinert, Cora Weigert, Francisco Garcia-Carrizo, Tim J. Schulz, Michael Ebner, Christian Freund, Annette Schürmann, Heike Vogel

**Affiliations:** 1 German Center for Diabetes Research (DZD e.V.), München-Neuherberg, Germany; 2 Department of Experimental Diabetology, German Institute of Human Nutrition Potsdam-Rehbruecke, Nuthetal, Germany; 3 Institute of Chemistry and Biochemistry, Protein Biochemistry, Freie Universität Berlin, Berlin, Germany; 4 Department of Molecular Physiology of Exercise and Nutrition, German Institute of Human Nutrition Potsdam-Rehbruecke, Nuthetal, Germany; 5 Institute of Nutritional Sciences, University of Potsdam, Nuthetal, Germany; 6 Department for Diagnostic Laboratory Medicine, Institute for Clinical Chemistry and Pathobiochemistry, University Hospital Tübingen, Tübingen, Germany; 7 Institute for Diabetes Research and Metabolic Diseases of the Helmholtz Zentrum München, University of Tübingen, Tübingen, Germany; 8 Department of Adipocyte Development and Nutrition, German Institute of Human Nutrition Potsdam-Rehbrücke, Nuthetal, Germany; 9 Institute of Molecular Biochemistry, Biocenter, Medical University of Innsbruck, Innsbruck, Austria

**Keywords:** Picalm, Myogenesis, Endocytosis, Skeletal muscle, Intermittent fasting, Exercise

## Abstract

**Objectives:**

Skeletal muscle is a central regulator of metabolic health, serving as the primary site of postprandial glucose uptake and playing a critical role in whole-body insulin sensitivity. Despite its importance, the molecular mechanisms governing muscle differentiation (myogenesis) and their modulation by metabolic interventions remain poorly defined. This study identifies the clathrin adaptor protein Picalm (phosphatidylinositol-binding clathrin assembly protein) as a novel regulator of myogenesis and investigates its regulation in response to exercise training and intermittent fasting.

**Methods:**

Functional characterization of Picalm was conducted in C2C12 myoblasts and primary myocytes using siRNA-mediated knockdown. Clathrin-mediated endocytosis was performed using dynamin inhibition (Dyngo-4a) and via an EGF internalization assay. Surface proteome alterations were analyzed by plasma membrane proteomics, and autophagy dynamics were assessed via immunoblotting and fluorescence imaging. Jasplakinolide was used to rescue differentiation defects by enhancing actin polymerization.

**Results:**

*Picalm*-depleted C2C12 myoblasts exhibited impaired differentiation, presumably due to diminished intracellular trafficking dynamics of cell surface proteins. Inhibition of dynamin-dependent endocytosis phenocopied the differentiation defect and further aggravated myogenesis in *Picalm*-depleted cells, indicating that Picalm-dependent endocytic function is required for efficient differentiation. Consistent with this, *Picalm* knockdown significantly decreased clathrin-dependent uptake of EGF. Proteome analysis of a plasma membrane-enriched fraction revealed increased abundance of over 100 proteins after *Picalm* knockdown, particularly candidates involved in vesicular trafficking (Vamp3, Vamp5), actin remodeling (Actn1, Actn4, Rhog, Rock1, Rock2) and cell adhesion (integrin receptors). In line with this, *Picalm* knockdown resulted in impaired maturation and lysosomal degradation of autophagic vesicles. Remarkably, pharmacological stabilization of actin filaments with Jasplakinolide restored myogenic differentiation in *Picalm*-deficient cells, highlighting a functional link between actin remodeling and myogenesis.

**Conclusions:**

Picalm regulates skeletal muscle differentiation by supporting clathrin-mediated endocytosis and plasma membrane remodeling, thereby maintaining trafficking-dependent control of actin organization. Its expression is responsive to metabolic cues such as exercise and intermittent fasting. These findings reveal a novel molecular link between nutrient signaling and myogenesis, with implications for metabolic disease and muscle regeneration.

## Highlights

• Picalm is a lifestyle-responsive regulator of myogenesis, upregulated in muscle after exercise and intermittent fasting.
• Loss of *Picalm* impairs myogenesis by disrupting endocytosis, trafficking, surface proteins and actin dynamics.
• Pharmacological stabilization of actin filaments rescues the differentiation defect in *Picalm*-deficient myoblasts.

## 1 Introduction

Skeletal muscle plays a pivotal role in metabolic health, acting as the main site of postprandial glucose disposal and thus maintaining whole-body glucose homeostasis [1]. It is uniquely capable of responding to exercise-induced contraction with an immediate increase in insulin sensitivity and secretion of myokines [2,3]. Pathophysiological changes of skeletal muscle metabolism associated with obesity and/or loss of muscle mass in the context of sarcopenic obesity can increase the risk for developing type 2 diabetes (T2D) [4].

In this study, we aimed to uncover common molecular determinants of skeletal muscle health that respond to both exercise and diet-based interventions for T2D prevention in an obese and diabetes-prone mouse model. Thereby, *Picalm* (phosphatidylinositol-binding clathrin assembly protein) was identified as a novel lifestyle-responsive gene in skeletal muscle. Picalm is a ubiquitously expressed clathrin-adaptor protein mainly studied in the context of neuronal function, as several single nucleotide polymorphisms (SNPs) within this gene have been associated with Alzheimer's disease [5]. Hence, numerous studies have focused on the role of Picalm in the processing of amyloid β plaques and the formation of pathological tau aggregates [[6], [7], [8], [9]]. Under normal physiological conditions, Picalm is involved in clathrin-mediated endocytosis and regulates the sorting of R-SNARE proteins (soluble NSF attachment protein receptors), which are crucial for synaptic vesicle fusion and other vesicular processes such as autophagy [10,11]. Picalm is therefore implicated in neurotransmission and the endo-lysosomal system. However, research on Picalm biology in non-neuronal tissues remains limited, and conflicting findings from various *in vitro* and *in vivo* systems - such as regarding the effect of Picalm knockdown on transferrin receptor uptake [[12], [13], [14], [15]] - suggest that Picalm may have cell type-specific functions. No studies have yet investigated the functional role of Picalm in skeletal muscle or muscle cells *in vitro*.

Our previous research identified *Picalm* as being associated with obesity and T2D in adipose tissue, highlighting its role in insulin signaling and GLUT4 translocation in adipocytes [16]. In the current study, we investigated the function of Picalm in muscle cell biology using C2C12 myoblasts.

We discovered that *Picalm* knockdown resulted in severe myogenesis defects, potentially evoked by an interplay of disrupted actin dynamics - due to impaired sorting of Ras- and Rho-related GTPases and other modulators of the cytoskeleton.

## 2 Results

### 2.1 Picalm expression in skeletal muscle increases following fasting- and exercise-based lifestyle interventions

**Figure 1:**
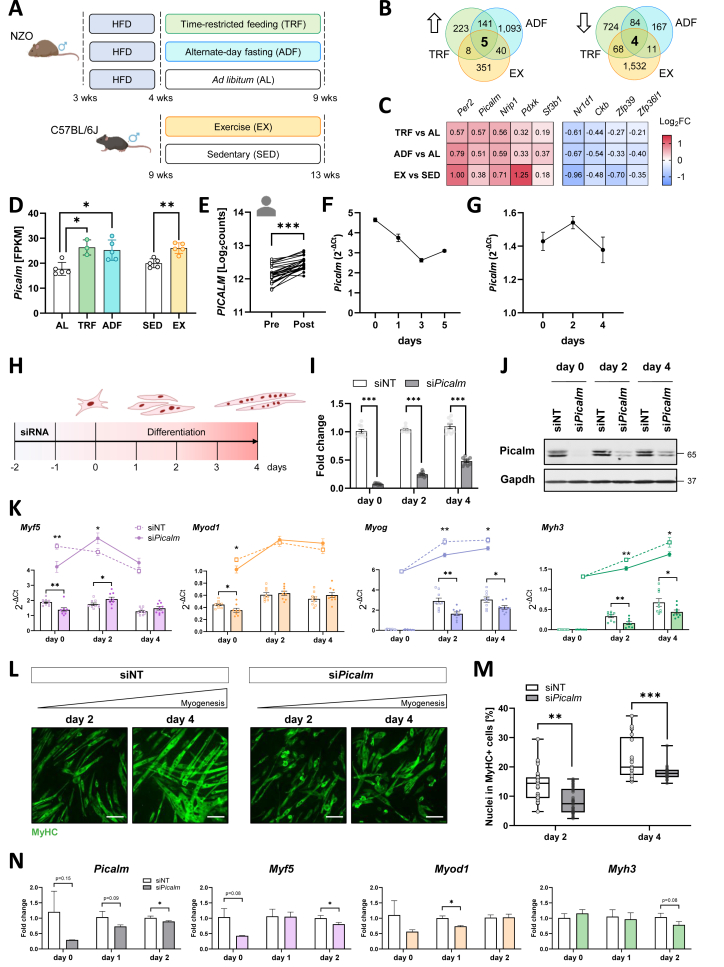
***Picalm* is regulated by intermittent fasting and exercise and is essential for myogenesis.** (A) NZO mice were fed a high-fat diet (HFD) and assigned to time-restricted feeding (TRF), alternate-day fasting (ADF) or *ad libitum* (AL) feeding (n = 3–5/group); C57BL/6J mice were subjected to treadmill training (EX; SED, sedentary controls, n = 5/group). (B) RNA sequencing of quadriceps muscle revealed five commonly up- and four commonly down-regulated genes between TRF, ADF and EX. (C) Heatmap depicting Log_2_FC of commonly regulated genes. (D) *Picalm* expression in quadriceps of mice subjected to diet or exercise interventions and (E) in *vastus lateralis* of overweight or obese participants at baseline and after an ergometer exercise session (n = 25). (F) *Picalm* expression in primary murine myoblasts throughout differentiation (n = 3). (G) Analysis of *Picalm* expression during C2C12 cell differentiation, assessed by qRT-PCR (n = 3 independent experiments, each performed in triplicates). (H) Experimental setup for siRNA-mediated downregulation of *Picalm* in C2C12 cells prior to differentiation. (I) *Picalm* knockdown efficiency compared to cells treated with non-targeting siRNA (siNT), assessed by qRT-PCR (n = 3 independent experiments, each performed in triplicates) and (J) western blot. (K) Expression profiles of *Myf5*, *Myod1*, *Myog* and *Myh3* in si*Picalm* vs. siNT cells, analyzed by qRT-PCR (n = 3 independent experiments, each performed in triplicates). (L) Myoblast fusion was evaluated on days 2 and 4 by staining for myosin heavy chain (MyHC) and DAPI in si*Picalm* and siNT C2C12 cells (n = 3 independent experiments, each performed in duplicates or triplicates with 5 fields of view analyzed per individual replicate). Representative images for MyHC staining are shown. Scale bar: 100 μm. (M) Quantification of MyHC + cells. Percentage of nuclei in MyHC + cells based on the total nuclei count per image. (N) Expression profiles of *Picalm*, *Myf5*, *Myod1* and *Myh3* in si*Picalm* vs. siNT primary myocytes, analyzed by qRT-PCR (n = 2–3). ∗p < 0.05, ∗∗p < 0.01, ∗∗∗p < 0.001 by Student's t-test with Welch correction.

To identify novel regulators of skeletal muscle health, we analyzed RNA sequencing data from mice subjected to different lifestyle interventions. Specifically, the effects of time-restricted feeding (TRF) and alternate-day fasting (ADF) were studied in the obesity- and diabetes-prone New Zealand Obese (NZO) mouse model, which is protected from a diabetes-like phenotype by these interventions [17,18]. We performed transcriptomic analysis of quadriceps muscle at nine weeks of age (after five weeks of intervention), capturing early adaptations before manifestation of hyperglycemia in the *ad libitum*-fed group (AL) [17]. To identify common molecular signatures, we also analyzed skeletal muscle samples of C57BL/6J mice following four weeks of progressive treadmill training (EX) compared to a sedentary control group (SED) [19] (Fig. 1A).

Comparative analysis of the RNA sequencing data revealed nine genes with common expression changes after TRF, ADF and EX (Fig. 1B). These included key regulators of the circadian clock such as *Per2*, *Nrip1* and *Nr1d1*, aligning with previous reports highlighting their responsiveness to lifestyle interventions [[20], [21], [22], [23]], and regulators of transcription and splicing (*Sf3b1, Zfp39, Zfp36l1*) (Fig. 1C). Notably, *Picalm* (phosphatidylinositol-binding clathrin assembly protein), a gene we recently identified as a novel regulator of GLUT4 translocation in adipose tissue [16], was significantly upregulated in muscles of TRF and ADF compared to AL mice, as well as in EX compared to SED mice (Fig. 1D). This finding was further validated by transcriptome data from skeletal muscle of human subjects following an aerobic exercise session [24], which resulted in increased *PICALM* expression in all participants (Fig. 1E). We therefore aimed to elucidate the role of Picalm in lifestyle-induced improvements of skeletal muscle health and to identify the molecular mechanisms that regulate *Picalm* expression under these conditions.

To explore the role of Picalm in muscle, its expression pattern was first studied in muscle cells. In primary murine myoblasts, the expression of *Picalm* was highest prior to differentiation and gradually declined, reaching a stable plateau at day 3, hinting towards a function in the initial stages of muscle cell differentiation (Fig. 1F). As the continuous modulation of *Picalm* expression in primary myoblasts proved challenging, subsequent experiments were conducted in the murine myoblast cell line C2C12 to further examine Picalm's function. In these cells, Picalm expression was highest in the intermediate stages of differentiation (day 2, Fig. 1G).

### 2.2 *Picalm* knockdown significantly impairs myogenic capacity of C2C12 cells

Given Picalm's expression profile during *in vitro* myogenesis, we aimed to study the impact of sustained *Picalm* knockdown on C2C12 differentiation. Knockdown was performed in sub-confluent C2C12 myoblasts two days prior to the initiation of differentiation (day −2, Fig. 1H). This resulted in a marked reduction of *Picalm* mRNA expression by approximately 90%, 75% and 60% at days 0, 2 and 4 of differentiation, respectively, in si*Picalm*-treated cells compared to cells treated with non-targeting siRNA (siNT; Fig. 1I). A comparable reduction was also observed at the protein level (Fig. 1J). The knockdown of *Picalm* significantly disrupted the expression patterns of key myogenic regulatory factors (MRFs). The expression of the myogenic factor 5 (*Myf5*), an early determination factor important for myoblast expansion, was markedly reduced prior to differentiation (day 0), but increased above siNT levels by day 2 of differentiation. Similarly, other MRFs, including myogenic differentiation 1 (*Myod1*), myogenin (*Myog*) and the functional marker myosin 3 (*Myh3*), were significantly reduced in si*Picalm* compared to siNT cells (Fig. 1K). In line with these findings, si*Picalm* cells exhibited a significantly reduced capacity to form myosin heavy chain-positive (MyHC+) myotubes on days 2 and 4 of differentiation (Fig. 1L-M). This was accompanied by distinct morphological differences, as si*Picalm* cells displayed an irregular shape, in contrast to the elongated morphology in siNT cells (Fig. 1L). To extend the initial functional findings, myogenic progenitors were isolated from C57BL/6J mice, transfected with si*Picalm* or a control siRNA, differentiated into myotubes, and analyzed for the expression of representative myogenic markers at various stages of differentiation (Fig. 1N). Consistent with the observations in C2C12 cells, reduced *Picalm* expression impaired the myogenic program, although the effects were less pronounced due to high variability in knockdown efficiency. Overall, these results suggest that *Picalm* knockdown delays myoblast differentiation.

### 2.3 Myogenic differentiation depends on Picalm-dependent clathrin-mediated endocytosis

Various cellular processes involved in muscle cell development require the abundance of specific receptors, transporters and adhesion proteins at the cell surface [25]. Hence, efficient protein internalization and endocytosis are integral to proper myogenic differentiation. Since Picalm is a well-known regulator of clathrin-mediated endocytosis (CME), facilitating the formation of clathrin-coated pits through its interaction with clathrin and cargo-binding adaptor proteins like adaptor protein complex 2 (AP2) [10], we aimed to investigate if an impaired endocytosis mediates the dysregulated myogenesis.

**Figure 2:**
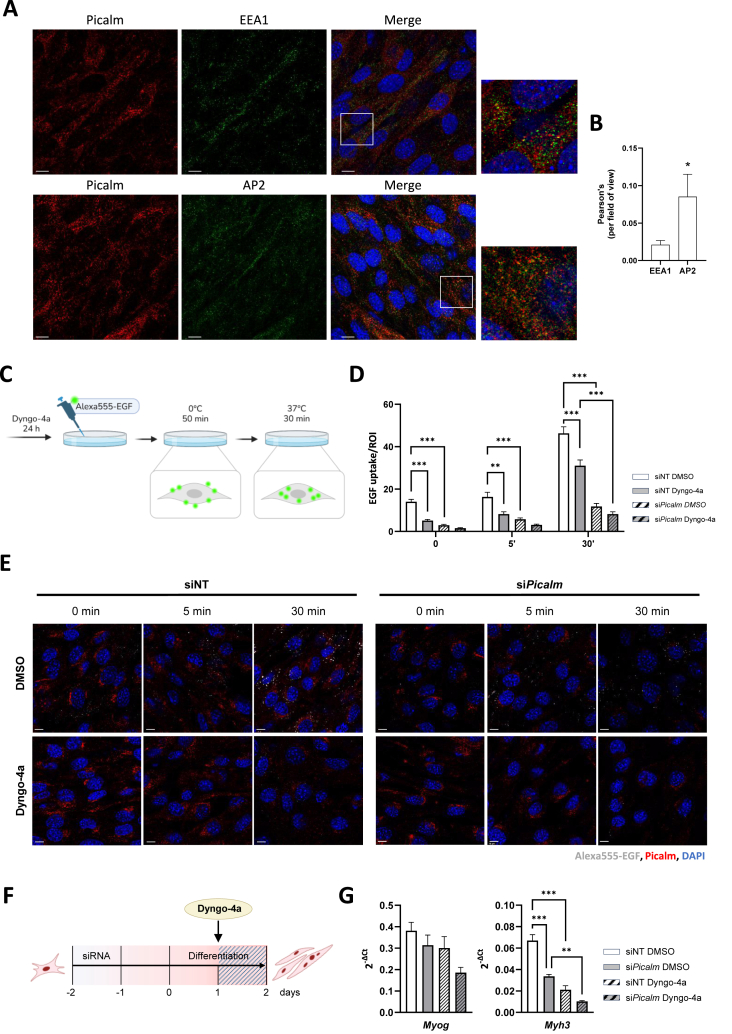
**Picalm knockdown impairs clathrin-mediated endocytosis in C2C12 cells.** (A) Co-localization of Picalm with the endosomal marker EEA1 and the AP2 adaptor complex in C2C12 cells (day 4); nuclei were stained using DAPI. Scale bar: 10 μm. (B) Pearson's correlation coefficient between fluorescent signal of Picalm (red) and EEA1 or AP2 (green) per field of view. (C) Fluorescence-labelled epidermal growth factor (Alexa555-EGF) was applied to serum-starved myoblasts (day 1) after treatment with the inhibitor Dyngo-4a for 24h. Surface binding was allowed at 0°C for 50 min, followed by washing to remove excess Alexa555-EGF and internalization was then permitted at 37°C for up to 30 min. After fixation, cells were subjected to immunofluorescent microscopy using an anti-Picalm antibody. (D) Quantification of internalized Alexa555-EGF intensity after 0, 5 and 30 min of incubation at 37°C. Intracellular signals were quantified by selecting regions of interest (ROI) in perinuclear regions (n = 3 independent experiments, 10–12 images per experiment, 12 ROI per image). (E) Representative images for EGF uptake assay. Scale bar: 10 μm. (F) The clathrin-mediated endocytosis (CME)-inhibitor Dyngo-4a was added to differentiation medium of C2C12 cells 24h after start of differentiation and incubated for 24h. (G) Expression profiles of *Myog* and *Myh3* in si*Picalm* vs. siNT cells following Dyngo-4a treatment analyzed by qRT-PCR (n = 2 independent experiments, each performed in dublicates). ∗p < 0.05, ∗∗p < 0.005, ∗∗∗p < 0.001.

First, to assess the association of Picalm with endocytic compartments in C2C12 cells, we performed co-staining with AP2 and EEA1 (early endosomal antigen 1). Both markers showed overlapping localization with Picalm at the plasma membrane and within intracellular compartments in both myoblasts (Supplementary Fig. 1a) and fully differentiated myotubes (Fig. 2A). Notably, Picalm displayed more prominent co-localization with AP2, consistent with its close association with clathrin-mediated endocytic sites (Fig. 2B and Supplementary Fig. 1b).

To assess whether *Picalm* depletion affects endocytosis, an epidermal growth factor (EGF) uptake assay was performed (Fig. 2C). In siNT control cells, EGF uptake increased progressively from 5 to 30 min, reflecting efficient receptor-mediated endocytosis, whereas si*Picalm* cells displayed significantly reduced uptake at all timepoints, indicating an impairment in growth factor internalization (Fig. 2D–E). Pharmacological inhibition of CME using Dyngo-4a, a specific inhibitor of dynamin-dependent membrane scission during endocytosis, markedly reduced EGF uptake in siNT cells and further decreased EGF internalization in si*Picalm* cells (Fig. 2D–E). This additive effect suggests that *Picalm* depletion may not completely abrogate CME, but rather reduce its efficiency, while residual dynamin-dependent vesicle scission remains functional and susceptible to pharmacological inhibition.

This graded impairment of CME was paralleled by a corresponding attenuation of myogenesis, consistent with a functional dependency. Specifically, Dyngo-4a treatment resulted in a significant reduction of myogenic capacity in siNT control cells, as assessed by the expression of the myogenic markers *Myh3* and *Myog* (Fig. 2F–G), phenocopying the differentiation defect observed upon *Picalm* knockdown. In line with the findings for EGF-uptake, Dyngo-4a treatment further aggravated the impaired myogenic capacity of si*Picalm* cells (Fig. 2G).

Together, these results support a model in which *Picalm* depletion partially compromises clathrin-mediated endocytosis, leading to reduced growth factor internalization and impaired myogenic differentiation. The response to dynamin inhibition indicates that the remaining endocytic activity in *Picalm*-deficient cells is still dynamin-dependent, consistent with a reduction rather than a complete blockade of CME efficiency.

**Figure 3:**
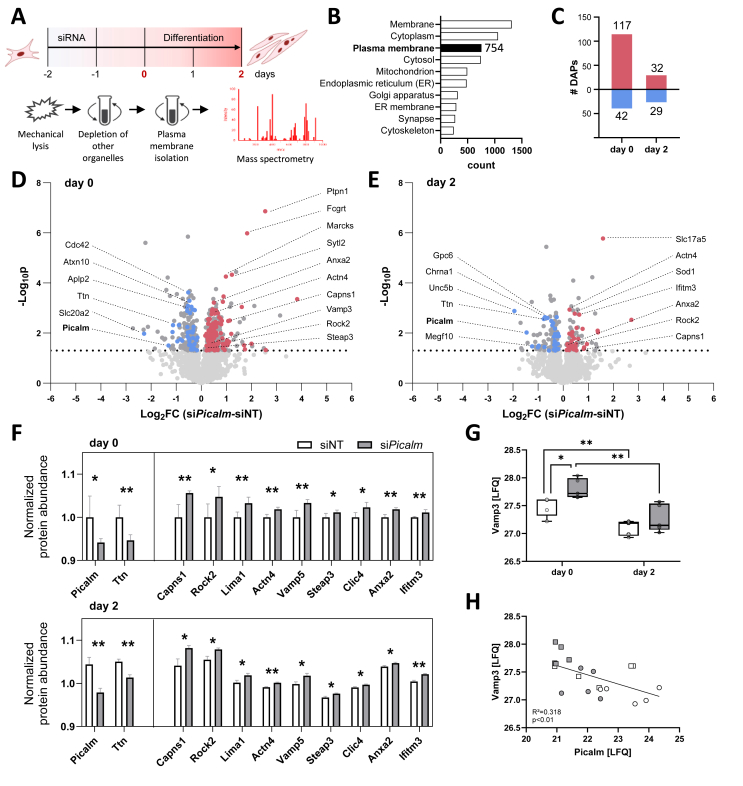
**Global changes in plasma membrane proteome and receptor abundance following *Picalm* knockdown.** (A) Schematic representation of the differential centrifugation protocol used to separate the plasma membrane from other cellular fractions in C2C12 cells. The PM-enriched fraction was obtained from day 0 and day 2 myoblasts treated with *Picalm*- or non-targeting siRNA (treated at day −2; n = 5/group). (B) Gene ontology - cellular compartment (GO-CC) enrichment analysis performed with total number of 2,212 quantified proteins. (C) Number of differentially abundant proteins (DAPs) between plasma membrane fractions of si*Picalm* and siNT cells at day 0 and day 2 (p < 0.05). (D) Distribution of proteins quantified by plasma membrane proteomics in si*Picalm* and siNT myoblasts at day 0 and (E) day 2. Red dots represent proteins more abundant upon knockdown of *Picalm* while blue dots represent proteins more abundant in siNT cells (p < 0.05) with GO-CC plasma membrane annotation. (F) Overlap of DAPs between si*Picalm* and siNT cells at day 0 and day 2. Normalized protein abundance relative to mean of siNT day 0 is shown for all commonly regulated proteins. (G) Plasma membrane Vamp3 abundance measured by quantitative mass spectrometry using label-free quantification (LFQ) and (H) correlation of plasma membrane abundance of Vamp3 to Picalm across conditions and timepoints (white symbols indicate siNT, grey symbols indicate si*Picalm*, squares indicate day 0, circles indicate day 2). ∗p < 0.05, ∗∗p < 0.01 by Student's t-test with Welch correction.

While endocytosis and myogenesis were functionally linked, the molecular intermediates connecting these processes remained undefined. We therefore examined which membrane proteins, apart from EGFR, are misregulated upon *Picalm* depletion and may drive the myogenic defect. Therefore, we performed an unbiased mass spectrometry-based quantitative proteome analysis of fractions enriched in plasma membrane (PM) proteins isolated from siNT and si*Picalm* homogenates (day 0 and day 2 of differentiation) using density-based fractionation (Fig. 3A, n = 5/group). Western blotting for Na^+^/K^+^ ATPase confirmed the enrichment of PM proteins in the obtained PM fraction (Supplementary Fig. 1c). In total 2,212 proteins were identified and quantified by mass spectrometry, including 754 classified as PM proteins based on gene ontology (GO) (Fig. 3B) (Supplementary Table 1). Overall for these PM-annotated proteins, more pronounced differences between si*Picalm* and siNT cells were observed prior to the onset of differentiation (day 0) compared to day 2, with 159 compared to 61 differentially abundant proteins (DAPs), respectively (Fig. 3C–E). For further analysis, only PM-annotated proteins - highlighted in blue and red in the volcano plots - were considered (Fig. 3D–E).

The majority of these DAPs were higher abundant at the PM under *Picalm*-depleted conditions, suggesting an impaired protein internalization in the absence of Picalm. Most of the changes observed prior to differentiation were not sustained on day 2, with the exception of Picalm and 10 other DAPs (Fig. 3F), including vesicle associated membrane protein 5 (Vamp5). It is well documented that Picalm serves as a specific adaptor protein for the internalization of Vamp proteins [26], which play important roles in vesicular trafficking processes including autophagy [27]. In addition to Vamp5, Vamp3 was also significantly upregulated at the PM of *Picalm*-depleted cells (day 0), as validated by immunocytochemistry, and significantly correlated with Picalm levels across conditions (Fig. 3G–H, Supplementary Fig. 1d).

### 2.4 *Picalm* depletion affects autophagy at intermediate to late stage

**Figure 4:**
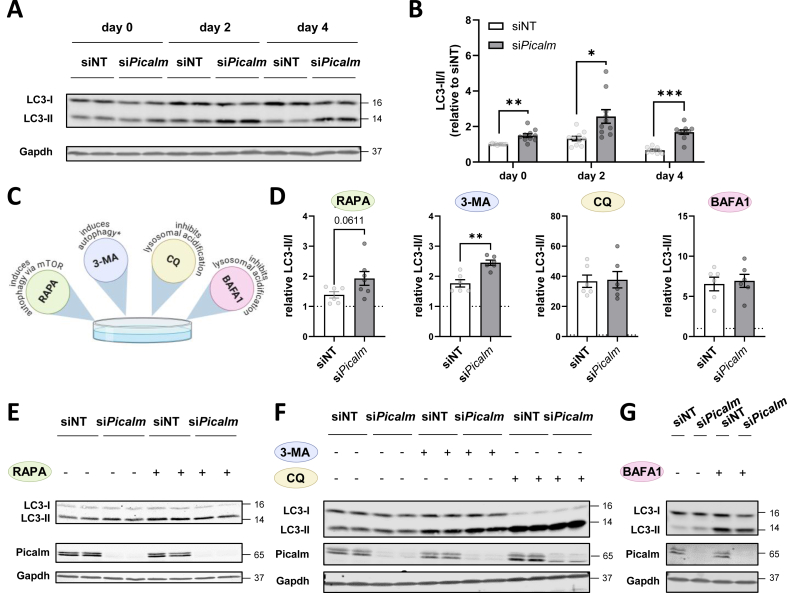
**Picalm is required for lysosomal degradation of autophagic vesicles.** (A) Western blot analysis of LC3-I and LC3-II protein levels in si*Picalm* and siNT C2C12 cells during differentiation. (B) Quantification of LC3-II/I ratio relative to levels of siNT at day 0, 2 and 4 (n = 3 independent experiments, performed in duplicates or triplicates). (C) Treatment with autophagic modulators (Rapamycin: RAPA; 3-Methyladenine: 3-MA; Chloroquine: CQ; Bafilomycin-A1: BAFA1) was performed on day 0 for 6h. ∗3-MA induces autophagy under nutrient-rich conditions but inhibits autophagy under nutrient-deprived conditions (n = 3 independent experiments, performed in duplicates or triplicates). (D) Quantification of LC3-II/I ratio normalized to the respective untreated siNT condition for each individual experiment. (E–G) Western blot analysis of LC3-II and LC3-I, and Gapdh as loading control. ∗p < 0.05, ∗∗p < 0.01, ∗∗∗p < 0.001 by Student's t-test with Welch correction.

Picalm itself is also known as cargo-binding adaptor protein for Vamps (vesicle associated membrane proteins) [26]. Increased Vamp3 and Vamp5 are supposed to enhance authophagy. In line with higher abundance of Vamp proteins at the PM after *Picalm* knockdown, we found a progressively elevated LC3-II/I ratio in si*Picalm* cells throughout differentiation (Fig. 4A–B). Treatment with the autophagy-inducing agents Rapamycin (RAPA) and 3-Methyladenine (3-MA), which enhance autophagic flux under nutrient-rich conditions [28], increased the LC3-II/I ratio in siNT cells, with an even more pronounced effect in si*Picalm* cells (p = 0.061 and p < 0.01, Fig. 4C–F). Treatment with Chloroquine (CQ) and Bafilomycin A1 (BAFA1), inhibitors of autolysosome formation and lysosomal degradation, effectively increased the LC3-II/I ratio in siNT and si*Picalm* cells and no significant differences were observed between the two groups (Fig. 4C–G). Consistent with the LC3 data, p62 also accumulated upon CQ treatment in both siNT and si*Picalm* cells, with no significant differences between groups (Supplementary Fig. 1e). This indicates that autophagy is blocked at the level of autophagosome fusion with lysosomes after depletion of *Picalm*. In other words, while *Picalm*-depleted myoblasts can effectively increase autophagosome formation, their maturation and/or lysosomal degradation of LC3-II-containing autophagosomes is impaired, resulting in LC3-II accumulation under conditions of enhanced autophagic flux.

When fusion of autophagosomes with lysosomes is insufficient to preserve cellular homeostasis, apoptosis is commonly induced. Accordingly, si*Picalm*-treated cells exhibited increased levels of cleaved caspase 3 at 24h and 48h after siRNA treatment (day −1 and day 0) in si*Picalm* compared to siNT cells. This effect did not persist after the switch to differentiation medium (day 2 and day 4; Supplementary Fig. 1f–g). It is unlikely that enhanced apoptosis alone accounts for the impaired differentiation, as the experimental setup, including a 48h growth period, ensured that the cells reached confluency before induction of differentiation. Proliferation rates, assessed by BrdU incorporation, showed no significant differences 24h and 48h post-siRNA treatment (day −1 and day 0), resembling the critical timeframe for reaching confluency prior to the onset of differentiation (Supplementary Fig. 1h–i).

### 2.5 *Picalm* depletion interferes with actin remodeling required for differentiation

**Figure 5:**
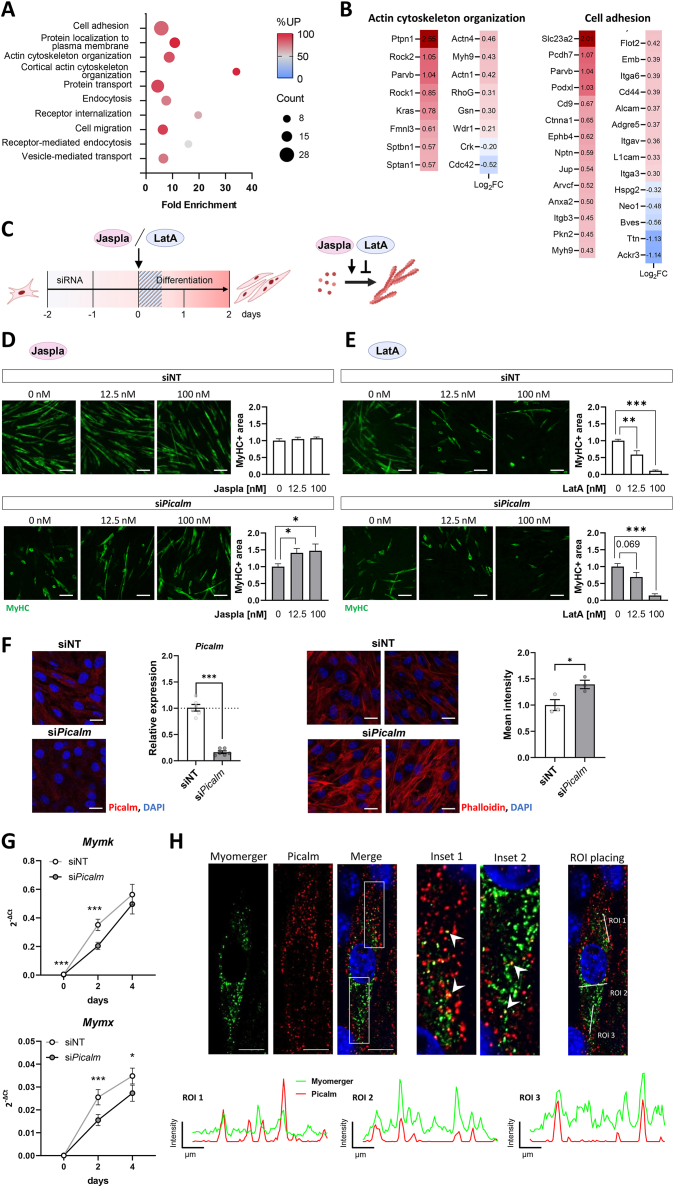
***Picalm* depletion impairs actin remodeling required for myoblast differentiation.** (A) Pathway enrichment analysis (gene ontology–biological processes; GO-BP) of the 159 plasma membrane differentially abundant proteins (DAPs) at day 0 measured by quantitative mass spectrometry. Dot size represents the count of DAPs within each term and color indicates the percentage of upregulated DAPs per term. (B) Log_2_FC of plasma membrane proteins involved in the indicated biological processes, which were enriched among all DAPs between *Picalm*-depleted and control C2C12 myoblasts before onset of differentiation (day 0). Log_2_FC indicates difference in abundance between si*Picalm* and siNT cells measured by quantitative mass spectrometry. (C) Experimental design: At the start of differentiation (day 0), siNT or si*Picalm* C2C12 cells were treated with either the actin polymerization inducer Jasplakinolide (Jaspla) for 6h or the actin polymerization inhibitor Latrunculin A (LatA) for 2.5h. Following treatment, the medium was replaced with fresh differentiation medium and cells were fixed on day 2 to evaluate their differentiation status. (D–E) Representative images and quantification of MyHC staining of cells treated without or with 12.5 or 100 nM of Jaspla or LatA, respectively and quantification of MyHC+ area relative to the untreated condition (n = 3 independent experiments, each performed in duplicates or triplicates with 5 fields of view evaluated per individual replicate). Scale bar: 100 μm. (F) Representative images of cells stained using anti-Picalm and DAPI (left side) as well as anti-Phalloidin and DAPI (right side). *Picalm* knockdown was quantified in si*Picalm* vs. siNT C2C12 cells (day 2 of differentiation) by qRT-PCR. (n = 3 independent experiments. Scale bar: 100 μm. (G) Expression levels of Myomaker (*Mymk*) and Myomerger (*Mymx*) throughout differentiation of C2C12 cells treated with non-targeting siRNA (siNT) or *Picalm*-targeting siRNA (si*Picalm*), measured by qRT-PCR (n = 3). (H) Co-localization of Picalm with Myomerger in differentiating C2C12 cells (day 2 of differentiation). Cells were stained using anti-Picalm and anti-Mymx antibodies and DAPI. Linear ROIs were placed to generate intensity profiles of Myomerger and Picalm. Scale bar: 10 μm. Arrow heads indicate co-localization. ∗p < 0.05, ∗∗p < 0.01, ∗∗∗p < 0.001 by Student's t-test with Welch correction.

Pathway enrichment analysis of all DAPs at the plasma membrane of si*Picalm* cells revealed an overrepresentation of proteins involved in (cortical) actin cytoskeleton organization, endocytosis and receptor internalization on day 0, and cell migration and muscle cell homeostasis on day 2 (Fig. 5A, Supplementary Fig. 2a). Based on the marked changes in the proteome of the PM-enriched fraction on day 0, we focused our analysis on this timepoint. As tight regulation of various signaling pathways is required for proper myogenesis, we screened our dataset for differentially abundant receptors. Although several receptors were higher abundant at the PM of si*Picalm* cells, this mainly affected cell adhesion receptors, such as several integrins (Itga3, Itga6, Itgav, Itgb3), rather than growth receptors which are more directly linked to cellular differentiation (Supplementary Fig. 2b). Specifically, the PM levels of Igf1r, Egfr, Fgfr1, Pdgfrb and Bmpr1a remained unchanged by *Picalm* knockdown (Supplementary Fig. 2c).

In addition to integrin receptors, numerous other cell adhesion molecules and actin remodeling regulators were upregulated at the PM of *Picalm*-depleted cells (Fig. 5B). Of note, *Picalm* knockdown upregulated the PM pool of the Rho GTPase RhoG and the Rho kinase 1 and 2 (Rock1 and Rock2; Fig. 5B), which are implicated in the formation of actin stress fibers and focal adhesions [29,30]. The same was observed for the actin crosslinking proteins alpha-actinin 1 and 4 (Actn1, Actn4; Fig. 5B). Notably, the upregulation of Rock2 and Actn4 was sustained on day 2 of differentiation (Fig. 3F).

To test whether disrupted actin dynamics may contribute to the impaired myogenic capacity, cells were treated with the actin polymerization promoting agent Jasplakinolide (Jaspla) or the inhibitor Latrunculin A (LatA). Given that the formation of an actin wall at cell–cell contact sites plays a crucial role in myoblast fusion [31], we treated the cells with these molecules. Treatment of the cells directly at the start of differentiation (Fig. 5C) with the actin stabilizing agent Jaspla rescued the differentiation defect of si*Picalm* cells dose-dependently (Fig. 5D). In contrast, treatment with LatA an inhibitor of actin polymerization interfered with differentiation in both siNT and si*Picalm* cells (Fig. 5E). This finding clearly indicates a causal link between dysregulated actin dynamics and diminished differentiation capacity of *Picalm*-depleted cells. To directly assess whether Picalm depletion alters F-actin organization, we performed phalloidin stainings in siNT and si*Picalm* cells at day 2 of differentiation. As shown in Fig. 5F, Picalm-depleted cells displayed stronger cortical phalloidin staining at day 2, indicative of increased F-actin bundling at the cell periphery, along with disorganized stress fiber architecture compared to control cells. This phenotype supports our functional findings and is consistent with enhanced integrin abundance and impaired actin remodeling in si*Picalm* cells.

Lastly, although no enrichment of fusion proteins was detected in the PM fraction of si*Picalm* cells, it remains possible that Picalm is also involved in the internalization of critical fusogens for myoblast fusion like myomaker and myomerger (also called myomixer or minion) [[32], [33], [34], [35]]. Unfortunately, these proteins were not quantified in the proteome analysis, likely due to their small size, which limits the generation of distinct peptides upon digestion. *Picalm* knockdown significantly reduced myomaker (*Mymk*) and myomerger (*Mymx*) mRNA expression during differentiation (Fig. 5G), likely as a result of decreased expression of MRFs that regulate them (Fig. 1K). Given that myomerger facilitates membrane curvature and fusion pore formation during myoblast fusion via its membrane destabilizing properties, tight regulation of its PM-levels is required to avoid undesired membrane deformations [36]. Although it remains unclear whether Picalm directly regulates its endocytosis, co-staining revealed that Picalm co-localizes with myomerger in intracellular puncta in untreated C2C12 myoblasts (Fig. 5H). Further experiments are needed to validate this potential interaction and explore its functional implications.

Taken together, these findings suggest that the myogenic defects observed in *Picalm*-knockdown cells may result from alterations in the PM proteome already before the onset of differentiation. *Picalm* depletion in C2C12 myoblasts resulted in an accumulation of cell adhesion and actin regulators at the plasma membrane, involving numerous GTPases, along with changes in the levels of key receptors required for myogenesis. These disruptions likely hinder the dynamic morphological changes essential for progressing to later stages of differentiation, such as elongation and syncytia formation.

### 2.6 *Picalm* knockdown does not affect mitochondrial capacity

As intermittent fasting and exercise, conditions enhancing *Picalm* expression in skeletal muscle (Fig. 1), improve metabolism and mitochondrial function, we assessed mitochondrial function before, during and after differentiation in control and si*Picalm* cells. No significant differences in basal, ATP-linked or maximal respiration or spare capacity were found between siNT and si*Picalm* cells at any timepoint (Supplementary Fig. 3a–e). Consistent with this, mitochondrial DNA content was unchanged between siNT and si*Picalm* cells across all timepoints (Supplementary Fig. 3f), suggesting that the abundance of functional mitochondria is unaffected in *Picalm*-depleted cells. To assess whether *Picalm* depletion affects insulin signaling in mature myotubes, we performed siRNA-mediated knockdown in fully differentiated myotubes (day 4), followed by insulin stimulation 48h later (day 6, Supplementary Fig. 3g). Compared to siNT-treated cells, this resulted in an approximately 30% reduction in Picalm protein level (Supplementary Fig. 3h–i). In contrast to our observations in adipocytes [16], no differences in insulin-stimulated Akt phosphorylation were detected between siNT and si*Picalm* myotubes across various insulin concentrations (Supplementary Fig. 3i–j).

## 3 Discussion

The positive effects of lifestyle interventions on metabolic health are well-documented [[37], [38], [39]]. However, their impact on skeletal muscle (SM), especially under fasting-based interventions, remains underexplored. Here, we identified *Picalm* as a novel fasting- and exercise-responsive gene in SM, which plays an essential role in myogenesis. *Picalm* depletion in myoblasts markedly impaired differentiation, pointing to a central role in membrane trafficking and cytoskeletal organization required for efficient muscle formation.

Our observations of elevated *Picalm* expression after exercise training in both mice and humans align with findings from Kleinert et al., they reported increased Picalm levels in SM of mice after exercise training on a high-fat diet [40] and a meta-analysis of human transcriptome datasets, revealing higher *PICALM* expression following acute aerobic or resistance exercise [41]. Additionally, elevated *Picalm* levels in SM following 3 days of cold exposure in mice [42] suggest that muscle contraction, such as shivering, may contribute to *Picalm* regulation.

Picalm's involvement in erythroid maturation [13] and adipogenesis [16] underscore its broader role in cellular differentiation across multiple lineages. However, evidence from Duchenne muscular dystrophy (DMD) indicates a muscle-specific role for Picalm, as its expression is markedly reduced in both DMD patients and animal models [43]. In line with this, our findings suggest that Picalm influences myogenesis through multiple mechanisms, primarily linked to its function within the endo-lysosomal system.

Picalm primarily functions within the endo-lysosomal system, contributing to clathrin assembly [10,44,45] and as specific adaptor for SNARE proteins [26]. Accordingly, *Picalm* knockdown resulted in the accumulation of more than 100 proteins in the plasma membrane-enriched fraction, presumably due to an impaired endocytic internalization and recycling. Notably, this fraction represents a plasma membrane-enriched preparation rather than a strictly purified plasma membrane fraction. Therefore, the impaired myogenic differentiation observed after *Picalm* depletion is unlikely to be explained solely by changes in individual surface receptors. Several of the affected proteins are annotated to endosomal or cytosolic compartments, reinforcing the notion that Picalm regulates interconnected trafficking pathways. Importantly, the remaining endocytic activity in *Picalm*-deficient cells remained dynamin-dependent, indicating reduced rather than completely abolished clathrin-mediated endocytosis. Thus, impaired myogenic differentiation following *Picalm* depletion likely arises from global disturbances in membrane trafficking dynamics.

One of the most striking findings was the altered PM-abundance of Rho GTPases and other proteins involved in actin remodeling upon *Picalm* depletion. These molecular switches control various cellular processes - including signal transduction and cytoskeletal organization - by cycling between an inactive GDP-bound and an active GTP-bound state that triggers downstream effector proteins [46]; and membrane association is often essential for their activation [47]. We observed that early treatment with the actin polymerization-promoting compound Jasplakinolide rescued the myogenesis defect in *Picalm*-depleted cells. This suggests that *Picalm* depletion causes disorganized rather than simply hyperstabilized actin structures, disrupting alignment and fusion. Consistent with this, phalloidin staining revealed increased cortical F-actin bundling and disorganized stress fiber architecture in si*Picalm* cells, indicative of excessive actin stabilization and reduced cytoskeletal plasticity. Previous reports have also shown that *Picalm* expression is upregulated in neuronal cells following exposure to actin-disrupting agents [48]. While our findings strongly indicate that Picalm is required for proper actin remodeling, the precise mechanism remains unclear. It is likely that Picalm regulates actin dynamics indirectly by controlling the plasma membrane localization of critical actin regulators, rather than through direct involvement in actin remodeling. This is supported by previous studies demonstrating the detrimental impact of impaired endocytosis on cytoskeleton organization [49,50]. It has been shown that clathrin is essential for actin scaffolding in C2C12 cells, with knockdown of either clathrin heavy chain or the adaptor protein complex AP2 disrupting cytoskeleton organization [51]. Additionally, loss of clathrin heavy chain impairs the formation of actin-based protrusions in C2C12 cells, resulting in defective myogenesis [52].

Beyond actin regulators, *Picalm* knockdown affected several other proteins and receptors implicated in myogenesis. As mentioned previously, integrin receptors - responsible for linking the cytoskeleton to the extracellular matrix and initiating intracellular signaling [53] - were more abundant at the PM in *Picalm*-depleted myoblasts. Excessive integrin activation has been shown to interfere with normal myogenesis by enhancing cell adhesion beyond optimal levels [54]. Moreover, integrin signaling significantly contributes to the regulation of myogenic regulatory factors [55,56], which were also found to be dysregulated following *Picalm* depletion. In addition, altered actin remodeling following *Picalm* depletion may secondarily influence integrin receptor localization and signaling. Given the tight interplay between the actin cytoskeleton, integrin-mediated adhesion complexes and membrane organization, disturbances in cytoskeletal dynamics could influence membrane fluidity and receptor mobility. While our data do not directly address membrane biophysical properties, this represents an interesting conceptual framework that warrants further investigation to better understand how *Picalm*-dependent trafficking may coordinate cytoskeletal remodeling with integrin signaling and membrane organization during myogenesis.

*Picalm* knockdown also affected SNARE proteins, particularly Vamp3 and Vamp5. In line with this, *Picalm*-depleted cells displayed impaired autophagosome formation - a Vamp3-dependent process critical for muscle development [9,57]. Notably, a direct interaction between Picalm and Vamp5 has not been previously reported, and its significance for myogenesis remains unclear. Earlier studies suggest that Vamp5, initially identified as a muscle-specific isoform known as myobrevin, is implicated in myogenesis [[58], [59], [60]]. However, apart from its localization to the ends of myotubes [59] and its progressively increasing expression pattern during *in vitro* differentiation [60], its specific function in this context remains poorly understood.

Additionally, we observed intracellular co-localization of Picalm and myomerger in C2C12 myoblasts, suggesting a possible role in trafficking proteins involved in myoblast fusion, such as myomaker and myomerger [32,34,51]. Since these fusion proteins transition from intracellular vesicles to the plasma membrane, Picalm may facilitate their proper localization during differentiation.

Interestingly, as intermittent fasting and exersice are both well known to improve insulin sensitivity and mitochondrial function, it was surprising to observe no changes in insulin response and oxygen consumption rate upon *Picalm* knockdown in mature myotubes, indicating that a Picalm-mediated improvement of myogenesis alone has no direct benefical effects on the metabolism. We can only speculate that a prolonged elevated Picalm expression in SM may lead to an improved insulin sensitivity and metabolism, when more muscle fibers developed. Alternatively, Picalm's role in muscle cells may be highly specific to cytoskeletal dynamics and differentiation processes rather than global metabolic regulation. Given Picalm's established function in adipocytes [16], where it has been implicated in insulin sensitivity and GLUT4 translocation, our results highlight a distinct context-dependent role in myogenic cells. This preservation of insulin responsiveness and mitochondrial function underscores that the differentiation defects observed are unlikely to be secondary to metabolic dysfunction and instead reflect a direct effect on actin remodeling and fusion machinery. Thus, our data delineate a selective requirement for Picalm in the structural and morphogenetic aspects of muscle formation, while leaving metabolic signaling pathways largely unaffected.

In summary, our study demonstrates that *Picalm* expression in skeletal muscle is regulated in response to lifestyle changes and likely contributes to myogenic capacity. Mechanistically, Picalm supports efficient endocytosis, which in turn ensures proper actin cytoskeletal remodeling, balanced integrin signaling, and coordinated SNARE-dependent trafficking and autophagy. Through fine-tuning intracellular trafficking dynamics, Picalm emerges as a critical regulator of the structural and morphogenetic processes underlying skeletal muscle formation.

## 4 Methods

### 4.1 Animal studies

#### 4.1.1 Time-restricted feeding and alternate-day fasting in NZO mice

Three-week-old male NZO/HIBomDife mice, from in-house breeding, were fed a high-fat diet (33% kcal fat, 49% kcal carbohydrate, 18% kcal protein, S8022-E080 unsat. FA; Ssniff, Germany) for one week before random assignment to the *ad libitum* (AL), time-restricted feeding (TRF) or alternate-day fasting (ADF) group, as previously described [17]. Before sacrificing, the feeding status of all groups was synchronized by a 6h fasting period.

#### 4.1.2 Exercise training

Nine-week-old male C57BL/6J mice (Charles River, Germany) were randomly assigned to the exercise (EX) or sedentary (SED) group, as previously described [19]. In brief, EX mice completed endurance exercise on a motorized treadmill (Exer6; Columbus Instruments, Columbus, OH, USA) on five subsequent days followed by two rest days for four weeks. Over time, the exercise intensity was progressively increased by adapting speed, incline and duration. The mice were sacrificed 3h after completion of the last training session.

All animals were kept in a 12:12h light–dark cycle at 22 ± 1°C and all animal experiments were approved by the ethics committee of the State Agency of Environment, Health and Consumer Protection (State of Brandenburg, Germany; ethics approvals: V3-2347-06-2011, V3-2347-23-2012, V3-2347-20-2013). All methods used in animal experiments were carried out in accordance with relevant guidelines, and handled according to standard use protocols and animal welfare regulations, and we also confirmed that all animal methods were reported in accordance with ARRIVE guidelines (http://arriveguidelines.org).

### 4.2 Human cohort

As previously described [24], 25 participants (64% female) with overweight or obesity and a sedentary lifestyle performed an 8-week endurance training intervention, consisting of 3 sessions per week of 30 min bicycle ergometer training and 30 min treadmill walking. All individuals provided detailed, informed written consent; the study protocol (NCT03151590) was approved by the ethics committee of the University of Tübingen and was in accordance with the Declaration of Helsinki.

*Vastus* *lateralis* biopsies used in this study were taken 8 days before start of the intervention and 60 min after the first ergometer exercise session. Gene expression analysis was performed using the Human Clariom S array (Thermo Fisher) as previously published [61].

### 4.3 Genome-wide transcriptome analysis

RNA was isolated from quadriceps using the RNeasy® Mini Kit (Qiagen) according to the manufacturer's instructions and RNA samples with RIN values ≥ 9 (Bioanalyzer, Agilent Technologies) were selected for Next Generation Sequencing. RNA sequencing (RNASeq) was conducted by BGI Group (Yantian District, Shenzhen, China) using the DNBseqTM sequencing platform, generating 30 million clean 150 base pairs reads per sample. The final library was generated using BGISEQ. Filtering of raw data after sequencing, including removing of adaptor sequences and low-quality reads, was performed by BGI Group. This included quality control by FastQC v0.11.9 and the read alignment to GRCm38.XX reference (Sanger database) using STAR version 2.7.9a. To normalize the raw reads, FPKM values (Fragments Per Kilobase per Million mapped fragments) were calculated. The calculation of differential expression was conducted using Student's t-test with Welch's correction.

### 4.4 Cultivation and differentiation of C2C12 cells

The murine myoblast cell line C2C12 was maintained in 75 cm^2^ cell culture flasks with high-glucose DMEM (Dulbecco's Modified Eagle Medium; DMEM, 4.5 g/L glucose, PAN-Biotech) supplemented with 10% fetal calf serum (FCS, Gibco) at 37°C and 5% CO_2_. Cells were passaged at 60% confluence using trypsin. For all experiments, except noted otherwise, cells were seeded in 24-well plates at a density of 55,000 cells per well. Upon reaching confluence, differentiation was induced by switching to high-glucose DMEM containing 2% horse serum (Gibco). The medium was changed every other day, until fully differentiated myotubes were formed on day 4 on differentiation medium.

For immunohistochemistry-based experiments, cells were seeded on coated coverslips, which were covered with serum-free DMEM (4.5 g/L glucose, PAN-Biotech) containing 1% extracellular matrix (Sigma–Aldrich) and 0.2% fibronectin (Sigma–Aldrich) and incubated at 37°C for at least 1h before cells were seeded.

### 4.5 siRNA transfection of C2C12 cells

For knockdown studies, cells were treated with murine small interfering RNA (siRNA, Picalm: L-041440-01-0020; non-targeting: D-001810-10-20; Horizon Discovery, Waterbeach, UK) targeted against the gene of interest, or non-targeting control siRNA, and Lipofectamine 2000 (Fisher Scientific). Per well (24-well-plate), 50 μL of each siRNA- (100 nM final concentration) and Lipofectamine-mix (1 μL/well) were separately prepared in serum-free DMEM (4.5 g/L glucose, PAN-Biotech), incubated at room temperature (RT) for 5 min, combined and incubated another 20 min at RT. Meanwhile, cells were washed with PBS, trypsinized (PAN-Biotech) and 55,000 cells per well were centrifuged at 1,000 rpm for 5 min (Biofuge 15R, Thermo Fisher Scientific). The cell pellet was resuspended in siRNA-Lipofectamine-mix and incubated at 37°C for 15 min, 400 μL serum-free DMEM (4.5 g/L glucose, PAN-Biotech) were added and 500 μL of the resulting cell suspension were seeded per well. After 5h, 500 μL of DMEM containing 20% FCS were added and 24h after start of transfection, the incubation with siRNA was ended by a change to culture medium. After another 24h, the cells received differentiation medium.

In order to investigate effects of *Picalm* knockdown in fully formed myotubes, transfection was performed after four days on differentiation medium. For 24-well plates, siRNA-Lipofectamine-mix was prepared as previously described and 100 μL were pipetted directly into the well containing 900 μL of fresh differentiation medium. For 96-well plates (used for Seahorse Assay), 7.5 μL of siRNA- and Lipofectamine-mix were prepared per well and 15 μL of the combined siRNA-Lipofectamine-mix were added to each well containing 135 μL fresh differentiation medium. To achieve effective knockdown, experiments were performed 48h after transfection.

### 4.6 Isolation of satellite cells for primary murine myoblast culture

For the isolation of mouse satellite cells from hind limb muscles and diaphragm of C57BL/6J mice the protocol by Gromova et al. [62] was followed with slight adaptations. Briefly, the collected tissues were minced in DMEM + 10% FCS (4.5 g/L glucose, PAN-Biotech), digested with 2.5 μg/μL Collagenase A (Roche) for 45 min at 37°C and Dispase II (2 units, Thermo Fisher) for 30 min at 37°C. After homogenization, filtering and centrifugation (300×*g*, 5 min) of the suspension, ACK lysis was performed in sorting medium (HBSS + 0.4% FCS). The pellet obtained after another centrifugation (300×*g*, 5 min) was dissolved in sorting medium, stained using specific antibodies (Table 1) for 30 min at 4°C and subsequently FACS-sorted (BD FACSMelody, BD Biosciences). The satellite cells were obtained from CD45-/CD31-/Sca1-/Itga7+ cell population, seeded into Matrigel-coated (Corning, New York, USA) wells and cultivated in DMEM + 10% FCS (4.5 g/L glucose, PAN-Biotech) supplemented with bFGF (5 ng/mL, Sigma) and Gentamycin (50 μg/mL, Sigma–Aldrich only applied directly after sorting). Differentiation was initiated upon establishment of close cell–cell contact (day 0) by switching to differentiation medium (DMEM, 1.0 g/L glucose, PAN-Biotech, supplemented with 2% horse serum, Gibco and Penicillin-Streptomycin (200 U/mL, Pan Biotech) and mature myotubes were formed after five days.

**Table 1:** Antibodies used for satellite cell isolation.

Antibody	Host species	Manufacturer	ID	Marker for
CD45-FITC	Rat	eBiocience	11-0451-82	Hematopoietic cells except mature erythrocytes and platelets
CD31-FITC	Rat	eBiocience	11-0311-82	Endothelial cells
Sca1-APC	Rat	eBiocience	17-5981-82	Fibro-adipogenic progenitor cells
Itga7-PE	Mouse	Miltenyi Biotec	130-103-355	Satellite cells

### 4.7 Primary myoblasts

Primary murine skeletal muscle cells were isolated from C57BL/6J mice based on a protocol published previously [63]. Briefly, dissected hind limb muscles were minced and enzymatically digested with collagenase type II (500 U/mL, Sigma), collagenase D (1.5 U/mL, Sigma), dispase II (2.5 U/mL, Sigma) and CaCl_2_ (2.5 mM, Roth) for 1h at 37°C. After centrifugation (300×*g*, 5 min), the suspension containing small pieces of the muscle tissue was resuspended in fresh proliferation medium, containing high-glucose DMEM (4.5 g/L glucose, PAN-Biotech), 20% fetal bovine serum (FBS, PAN-Biotech), 10% horse serum (PAN-Biotech), 0.5% chicken embryo extract (MP biomedicals), 2.5 ng/mL basic fibroblast growth factor (Sigma) and 1% penicillin/streptomycin (PAN-Biotech), and seeded on matrigel-coated (Corning, New York, USA) petri dishes. Cells were cultured at 37°C and 5% CO_2_. 48h before seeding the cells for experiments, cells were pre-plated on collagen type I-coated (Sigma) petri dishes to reduce the amount of non-myoblasts cells in the culture. For experiments, the cells were seeded on matrigel-coated (Corning, New York, USA) culture plates. The following day, myoblasts were transfected as described above. Subsequently, myoblasts were differentiated into myotubes using high-glucose DMEM containing 2% horse serum (PAN-Biotech) and 1% penicillin/streptomycin (PAN-Biotech), and harvested at the indicated timepoints.

### 4.8 Determination of cell proliferation by BrdU assay

The Bromodeoxyuridine (BrdU) assay was performed for quantitative assessment of cell proliferation rates. For this, cells were seeded on coverslips coated with ECM and were kept in culture medium until start of the experiment. The cells were incubated for 2h with BrdU (50 μM, Sigma–Aldrich) diluted in culture medium and subsequently washed with PBS, fixated with 4% para-formaldehyde (Roth) for 10 min, washed 3 times with PBS for 3 min. Finally, nuclei were stained using 4′,6-diamidine-2′-phenylindoledihydrochloride (DAPI; Roche), microscopy was performed and the number BrdU-positive nuclei was divided by the total number of nuclei as a measure of proliferative activity.

### 4.9 Treatment with autophagy modulators

All autophagy modulators were diluted in culture medium (DMEM, 4.5 g/L glucose, PAN-Biotech) and applied to C2C12 myoblasts (day 0) for 6h. To study the initial phase of autophagosome formation, Rapamycin (100 nM, Fisher Scientific) and 3-Methyladenine (5 mM, Sigma Aldrich) were used. To study the process of autolysosome formation and lysosomal degradation, Chloroquine (10 μM, Sigma Aldrich) and Bafilomycin A1 (400 nM, Biotechne) were used.

### 4.10 Dyngo-4a treatment and EGF endocytosis assay

C2C12 cells were treated with Dyngo-4a (Hydroxy-Dynasore) 24h after initiation of differentiation. Dyngo-4a was added to the differentiation medium at concentration of 80 μM and incubated for 24h. The myogenesis status was assessed 48h after initiation of differentiation via measuring the expression profile of the myogenic marker genes *Myog* and *Myh3* by qRT-PCR. For EGF endocytosis assay, 48h after initiation of differentiation, the cells were 2x washed with PBS, serum-deprived for 1h and incubated with 2 μg/mL Alexa555-coupled EGF (Alexa555-EGF, Invitrogen) on ice at 0°C for 50 min, washed 3 times with ice-cold PBS+10 mM MgCl before being transferred to 37°C for 0, 5 or 30 min in serum-free DMEM to allow internalization of surface-bound EGF. Cells were washed, fixated, subjected to immunocytochemistry, and ROI-based quantification of internalized EGF was carried out.

### 4.11 Treatment with actin polymerization modulators

Cells were treated with the actin modulating agents Latrunculin A (LatA) or Jasplakinolide (Jaspla) at the start of differentiation. LatA or Jaspla were added to the differentiation medium at concentrations of 12.5 nM or 100 nM and incubated for 2.5h (LatA) or 6h (Jaspla). The myogenesis status was assessed 48h after initiation of differentiation via MyHC staining.

### 4.12 Insulin stimulation assays

The insulin sensitivity of cells was evaluated by stimulation with insulin and analysis of downstream phosphorylation events. C2C12 cells (day 6 of differentiation) were first serum-depleted in Krebs-Ringer-Hepes-Buffer containing 0.1% bovine serum albumin (KRH-0.1%BSA) for 6h. Afterwards, cells were treated with insulin diluted to the indicated concentrations (5–100 nM) in KRH-0.1% BSA for 30 min at 37°C before cells were harvested. The ratio of Akt phosphorylation was determined by western blot.

### 4.13 Mitochondrial stress test

For assessing the mitochondrial function of C2C12 cells after *Picalm* knockdown, the Seahorse XF Cell Mito Stress Test Kit (Agilent Technologies) was used according to the manufacturer's instructions. This assay is based on the selective inhibition of complexes of the respiratory chain, allowing for the measurement of ATP-linked respiration, maximal respiration and non-mitochondrial respiration. This is achieved by a serial treatment with the compounds oligomycin, inhibiting the ATP synthase (complex V), the uncoupler Carbonyl cyanide-p-trifluoromethoxyphenylhydrazone (FCCP), disrupting ATP synthesis by interfering with the proton gradient along the mitochondrial membrane, and a mix of rotenone and antimycin A, inhibiting complex I and III to completely block mitochondrial respiration.

For this assay, C2C12 cells were transfected at a density of 12.500 cells per well (siNT) or 22,000 cells per well (si*Picalm*; 96-well Seahorse plate, Agilent Technologies). The cell count used for the si*Picalm* condition was increased in order to reach even levels of confluency between the conditions on the day after transfection, on which the differentiation process was initiated. Simultaneous measurement of cells on day 0, 2 and 4 on differentiation medium was achieved by staggered seeding of the cells on the same plate.

To investigate the effect of Picalm knockdown in fully differentiated myotubes, C2C12 cells were seeded at a density of 12,5000 cells per well (96-well Seahorse plate, Agilent Technologies) and the knockdown was performed four days after initiation of differentiation. Following an additional 48h incubation with the transfection agents, the Seahorse assay was conducted.

The cells were washed once and incubated at 37°C for 1h in 100 μL freshly prepared Seahorse medium (Table 2). Meanwhile, the prehydrated cartridge (Agilent Technologies) was loaded with the components (Table 3) and afterwards, the assay was started. In brief, the measurement consisted of four 18 min measurement cycles (3 × 3 min mix + 3 min measure) at baseline and after addition of the compounds from ports A-C (Table 3). Upon assay completion, 60 μL of RIPA buffer were added to each well and the plate was incubated on a Thermoshaker at 350 rpm and RT for 10 min. After centrifugation (4,000 rpm, 5 min, RT), BCA assay was performed.

**Table 2:** Composition of seahorse medium.

Component	Stock solution	Quantity	Final concentration	Manufacturer
XF base medium		98.32 mL		Agilent Technologies
d-glucose	2 mol/L	0.500 mL	10 mmol/L	Merck
Glutamine	200 mmol/L	1.00 mL	2 mmol/L	Sigma–Aldrich
Pyruvate	550 mmol/L	182 μL	1 mmol/L	Sigma–Aldrich
pH 7.4 (NaOH)

**Table 3:** Components used for preparation of the Seahorse cartridge (Agilent Technologies).

Component	Port	Concentration	Final concentration
Oligomycin	A	5 μM	1 μM
FCCP	B	12 μM	2 μM
Rotenone	C	3.5 μM	0.5 μM
Antimycin A	C	3.5 μM	0.5 μM
Diluted in seahorse medium (Table 2)			

### 4.14 Western blot analysis

Protein lysates were prepared from C2C12 cells or tissue samples and 5–15 μg of lysate were used for western blot analysis with specific antibodies (see Tables 4 and 5). For the detection of Picalm in combination with other proteins, two different Picalm antibodies from mouse and rabbit were used depending on the origin of the other used antibodies.

**Table 4:** Primary antibodies used for western blot analysis.

Target	Clonality	Host species	Manufacturer	ID
Picalm	Polyclonal	Rabbit	Sigma–Aldrich	HPA019053
Picalm	Monoclonal	Mouse	Santa Cruz	sc-271224
Phospho Akt (Ser473)	Polyclonal	Rabbit	Cell signaling	9271L
Akt	Monoclonal	Mouse	Cell signaling	2920
Gapdh	Polyclonal	Mouse	Ambion	AM4300
Caveolin-1	Polyclonal	Rabbit	Abcam	ab2910
Vamp5	Polyclonal	Rabbit	Synaptic systems	176003
Cleaved caspase 3	Polyclonal	Rabbit	Cell signaling	9661S
LC3	Polyclonal	Rabbit	Cell signaling	2775
Na+/K + -ATPase	Monoclonal	Mouse	Abcam	ab7671
p62	Polyclonal	Guinea Pig	Progen Biotechnik	GP62-C

**Table 5:** Secondary antibodies used for western blot analysis.

Target	Label	Host species	Manufacturer	ID
Anti-mouse	IRDye 680RD	Goat	LiCor	P/N 926-68070
Anti-mouse	IRDye 800CW	Donkey	LiCor	P/N 926-32212
Anti-rabbit	IRDye 680RD	Goat	LiCor	P/N 926-68071
Anti-rabbit	IRDye 800CW	Donkey	LiCor	P/N 926-32213

### 4.15 Real-time quantitative PCR (RT-qPCR)

For RT-qPCR, RNA was extracted from C2C12 cells or primary murine myoblasts using the RNeasy Mini kit (Qiagen) and cDNA was synthesized using M-MLV Reverse Transcriptase. The expression of target genes was analyzed using specific probe assays (Table 6) or specific primers with the SYBR Green method (Thermo Fisher, Table 7). The housekeeping genes *Tbp* and *Actin* were used for normalization.

**Table 6:** Probe assays used for RT-qPCR.

Gene	Fluorophore	Quencher	Probe	Manufacturer
*Picalm*	6-FAM	ZEN/IBFQ	Mm.PT.58.43171691	IDT
*Myf5*	6-FAM	ZEN/IBFQ	Mm.PT.58.5271235	IDT
*Myod1*	6-FAM	ZEN/IBFQ	Mm.PT.58.8193525	IDT
*Myog*	6-FAM	ZEN/IBFQ	Mm.PT.58.6732917	IDT
*Myh3*	6-FAM	ZEN/IBFQ	Mm.PT.58.32959978	IDT
*Tbp*	6-FAM	ZEN/IBFQ	Mm.PT.39a.22214839	IDT

**Table 7:** Primers used for SYBR Green RT-qPCR.

Gene	Primer sequences	
*Chdh1*	Forward: GCACAGTGGCCCTTAAATGT	Reverse: CCCTGCCTAAAATACCGTGA
*Cox2*	Forward: CCTGGTGAACTACGACTGCT	Reverse: GAATAACCCTGGTCGGTTTG
*Cox3*	Forward: GCAGGATTCTTCTGAGCGTTCT	Reverse: GTCAGCAGCCTCCTAGATCATGT
*mrRNA*	Forward: AGCCCATTTCTTCCCATTTC	Reverse: CGATAAACCCCGCTCTACCT
*Nd1*	Forward: GGATCCGAGCATCTTATCCA	Reverse: GGTGGTACTCCCGCTGTAAA
*Nd6*	Forward: ATTAAACAACCAACAAACCCAC	Reverse: TTTGGTTGGTTGTCTTGGGTT
*Mymk*	Forward: ATCGCTACCAAGAGGCGTT	Reverse: CACAGCACAGACAAACCAGG
*Mymx*	Forward: CTGAGCTGTCTGCTCTTTGT	Reverse: TCTCCTTCCTCTGGGAGTG
*Tbp*	Forward: TGTATCTACCGTGAATCTTGGC	Reverse: CCAGAACTGAAAATCAACGCAG
*β-Actin*	Forward: TACGAGCAGAGCCATACAG	Reverse: GCCAACCGTGAAAAGATGAC

### 4.16 Immunocytochemistry

C2C12 cells grown on coverslips coated with ECM were washed once with PBS and fixated with 4% para-formaldehyde/PBS for 10 min and washed with PBS (3 × 3 min). Residual non-crosslinked aldehyde-bonds were blocked with 50 mM NH_4_Cl/PBS (3 × 3 min), cells were permeabilized with 0.2% Saponine/PBS (6 min), excess Saponine was washed off with 0.02% Saponine/PBS (5 min) and unspecific binding sites were blocked with 2% BSA diluted in 0.02% Saponine/PBS (1h). Primary antibodies were diluted in blocking solution (Table 8) and incubated for 1h at RT or overnight at 4°C, cells were washed with 0.02% Saponine/PBS (3 × 10 min) and secondary antibodies (Table 9) diluted in blocking solution were applied for 1h at RT. All antibodies were applied individually and sequentially. Coverslips were mounted on microscope slides using Mounting Medium (Vectashield). For phalloidin staining, cells were incubated overnight at 4°C with Phalloidin-647 Alexa Fluor Plus (Invitrogen, A30107; 1:1,000) following the blocking step. Subsequently, nuclei were counterstained with DAPI (Roche, 10236276001) for 1h at RT.

**Table 8:** Primary antibodies used for immunocytochemistry.

Target	Clonality	Host species	Manufacturer	ID
Picalm	Polyclonal	Rabbit	Sigma–Aldrich	HPA019053
Picalm	Monoclonal	Mouse	Santa Cruz	sc-271224
Mymx (ESPG)	Polyclonal	Sheep	Invitrogen	PA5-47639
EEA1	Monoclonal	Mouse	Santa Cruz	sc-137130
Adaptin alpha (subunit of AP-2)	Monoclonal	Mouse	BD transduction lab	610501
Vamp3	Polyclonal	Rabbit	Synaptic systems	104103
Myosin heavy chain, sarcomere (MyHC)	Monoclonal	Mouse	DHSB	MF20

**Table 9:** Secondary antibodies used for immunocytochemistry.

Target species	Label	Host species	Manufacturer	ID
Mouse	Alexa-488	Goat	Fisher scientific	A11017
Mouse	Alexa-546	Goat	Fisher scientific	A11018
Mouse	Alexa-647	Donkey	Fisher scientific	A31571
Rabbit	Alexa-488	Goat	Fisher scientific	A11070
Rabbit	Alexa-546	Goat	Fisher scientific	A11035
Rabbit	Alexa-647	Donkey	Fisher scientific	A31573
Rat	Alexa-546	Goat	Fisher scientific	A11081
Sheep	Alexa-546	Donkey	Fisher scientific	A21098

### 4.17 Phalloidin staining

CellProfiler 4.2.6 was used to quantify phalloidin staining intensities. The applied pipeline contained the modules IdentifyPrimaryObjects to identify phalloidin positive areas, SplitOrMergeObjects to merge all phalloidin-positive objects into one, and MeasureObjectIntensity to calculate the average pixel intensity within the phalloidin-positive area. Values were normalized to NT control.

### 4.18 Colocalization Analysis of Picalm with EEA1 and AP2

Calculation of Pearson's correlation coefficient was carried out in Fiji/image j using the JACoP plugin [64]. Camera background was subtracted in all images equally by linear subtraction and Pearson's correlation coefficient was determined per field of view.

### 4.19 Plasma Membrane Proteome analysis

Differential ultracentrifugation with sucrose gradient purification was employed in order to fractionate cellular membranes into the following compartments: high-density microsomes (HDM), low-density microsomes (LDM) and plasma membrane (PM). The residual proteins were fractionated into the mitochondria and nuclei fraction and the cytosol. For this, siNT and si*Picalm* C2C12 myoblasts (day 0) grown in 24-well plates were washed with TES (Tris–EDTA-Sucrose buffer, Table 10) and a cell suspension was obtained by manual cell scraping using TES + PMSF (Phenylmethylsulfonylfluorid, 0.2 mM) and subsequent homogenization using a Potter-Elvehjem homogenizer (Thermo Scientific). Ultracentrifugation (7,000×*g* for 15 min, Optima MAX-TL, Beckman Coulter) was performed to deplete cytosolic proteins, LDM-membranes (primarily Golgi-membranes), HDM-membranes (primarily ER and endosomal membranes), which remained in the supernatant. This supernatant was further centrifuged (21,728×*g* for 45 min) to obtain the HDM fraction as a pellet and the residual supernatant was centrifuged (200,000×*g* for 90 min) to obtain the LDM fraction as a pellet and cytosolic proteins in the supernatant. The resulting pellet from the initial centrifugation was resuspended in TES + PMSF and homogenized using a Potter-Elvehjem homogenizer. The resulting suspension was transferred on top of a sucrose cushion (1.12 mol/L in TES) and centrifuged (100.000×*g* for 75 min) to obtain the mitochondria and nuclei fraction as a pellet. The PM was obtained on top of the sucrose cushion, dissolved in TED + PMSF and obtained as a pellet after another centrifugation step (100.000×*g* for 60 min).

**Table 10:** Composition of the Tris -EDTA-Sucrose buffer (TES).

Tris–HCL	20 mmol/L	Roth
EDTA	1 mmol/L	Roth
Sucrose	8.7%	Sigma
Protease inhibitor	1 tablet/50 mL	Roche
pH 7.4		

The obtained PM fractions were prepared for proteome analysis using the iST Sample Preparation Kit (Preomics). Therefore, the pellets were dissolved in lysis buffer (Preomics) and protein concentrations were measured with the Bicinchoninic acid assay (BCA-assay). The PM fractions were diluted in lysis buffer (7 μg/20 μL) and deglycosylation was performed by incubation with 1 μL PNGase F (Glycerol-free, New England Biolabs) at 37°C for 24h. Thereafter, sample preparation was continued using the iST Sample Preparation Kit according to the manufacturers protocol.

### 4.20 Nano liquid chromatography-mass spectrometry (LC-MS) and data analysis

Peptides were reconstituted in 0.05% trifluoroacetic acid, 4% acetonitrile in water, and peptide concentration was determined based on the sample absorbance at 205 nm using a NanoDrop spectrophotometer (Thermo Fisher Scientific). About 2 μg per sample were then analyzed by a Ultimate 3000 reversed-phase capillary nano liquid chromatography system connected to a Q Exactive HF mass spectrometer (Thermo Fisher Scientific). Samples were injected and concentrated on a trap column (PepMap100C18, 3 μm, 100 Å, 75 μm i.d. x 2 cm, Thermo Fisher Scientific) equilibrated with 0.05% TFA in water. After switching the trap column inline, LC separations were performed on a capillary column (double nanoViper PepMap Neo C18, 2 μm, 100 Å, 75 μm i.d. x 50 cm, Thermo Fisher Scientific) at an eluent flow rate of 300 nL/min. Mobile phase A contained 0.1% formic acid in water, and mobile phase B contained 0.1% formic acid in 80% acetonitrile/20% water. The column was pre-equilibrated with 5% mobile phase B followed by an increase of 5–44% mobile phase B in 100 min. Mass spectra were acquired in a data-dependent mode utilising a single MS survey scan (*m*/*z* 300–1650) with a resolution of 60,000, and MS/MS scans of the 15 most intense precursor ions with a resolution of 15,000 with a normalized collision energy of 27. The dynamic exclusion time was set to 20 s and automatic gain control was set to 3 × 10^6^ and 1 × 10^5^ for MS and MS/MS scans, respectively.

MS and MS/MS raw data were analyzed using the MaxQuant software package (version 2.5.2.0) with implemented Andromeda peptide search engine [65]. Data were searched against the reference proteome of *Mus musculus* downloaded from Uniprot (54,910 proteins, Proteome ID UP000000589). The default parameters in MaxQuant were used except for enabling the options label-free *quantification (LFQ) and match between runs.* Filtering and statistical analysis was carried out using the software Perseus version 2.0.11 [66]. Only proteins which were identified with LFQ intensity values in at least 3 out of 5 replicates (within at least one of the experimental groups) were used for downstream analysis. Missing values were replaced from normal distribution (imputation) using the default settings (width 0.3, down shift 1.8). Mean log2 fold protein LFQ intensity differences (Log_2_FC) between experimental groups were calculated in Perseus using Student's t-tests using the p value < 0.05 as a cut-off. Volcano plots were created by plotting the -log_10_ p-value against the mean log_2_ fold protein LFQ intensity differences.

## CRediT authorship contribution statement

**Jasmin Gaugel:** Data curation, Investigation, Writing – original draft. **Neele Haacke:** Data curation, Investigation. **Benno Kuropka:** Data curation, Investigation. **Markus Jähnert:** Methodology, Software. **Julia Rominger:** Data curation, Investigation. **Wenke Jonas:** Data curation, Investigation. **Thilo Speckmann:** Data curation, Investigation. **Niclas Rausch:** Data curation, Investigation. **Maximilian Kleinert:** Resources. **Cora Weigert:** Resources. **Francisco Garcia-Carrizo:** Data curation, Investigation. **Tim J. Schulz:** Resources. **Michael Ebner:** Methodology. **Christian Freund:** Resources. **Annette Schürmann:** Conceptualization, Supervision, Writing – review & editing. **Heike Vogel:** Conceptualization, Data curation, Funding acquisition, Investigation, Project administration, Supervision, Writing – original draft, Writing – review & editing.

## Funding

AS received funding from the German Ministry of Research, Technology and Space (DZD, Grant: 82DZD00302, 82DZD03D03, 82DZD03I04), and the State of Brandenburg. AS and HV received funding from the European Union’s Horizon Europe Research and Innovation Programme (OBELISK grant agreement 101080465).

## Declaration of competing interest

The authors declare that they have no known competing financial interests or personal relationships that could have appeared to influence the work reported in this paper.

## Data Availability

Data will be made available on request.

## Appendix A Supplementary data

Multimedia component 1 **Supplementary Figure 1: Adaptations of the Plasma Membrane Proteome in Response to *Picalm* Knockdown.** (a) Co-localization of *Picalm* with the endosomal marker EEA1 and the AP2 adaptor complex in C2C12 cells (day 2); nuclei were stained using DAPI. Scale bar: 10 µm. (b) Pearson‘s correlation coefficient between fluorescent signal of *Picalm* (red) and EEA1 or AP2 (green) per field of view. (c) Enrichment of the plasma membrane marker Na+/K+ ATPase in the plasma membrane-enriched fraction (PM) of C2C12 cells (day 0) retrieved by differential ultracentrifugation compared to high density microsomes (HDM) fraction and low density microsomes (LDM). (d) Subcellular localization of Vamp3 in siNT and si*Picalm* myoblasts: 48h after siRNA treatment, C2C12 myoblasts were fixated and stained using Vamp3- and *Picalm*-targeting antibodies, DAPI and Phalloidin to visualize f-actin. Scale bar: 10 µm. (e) Quantification of p62 by western blot analysis of cells treated with the autophagic modulator Chloroquine (CQ) performed on day 0 for 6h (n=3 independent experiments, performed in duplicates or dublicates). (f) Western blot analysis of cleaved caspase 3 levels in si*Picalm* and siNT C2C12 cells during differentiation, normalized to the housekeeping protein Gapdh. (g) Quantification of relative cleaved caspase 3 levels normalized to Gapdh, expressed relative to siNT levels (n=3 independent experiments, performed in duplicates or triplicates). (h) Proliferation capacity measured on day -1, day 0 and day 2 by BrdU incorporation assay. BrdU (50 µM) was applied to C2C12 cells for 2h before fixation of nuclei staining (DAPI). Representative images for day -1 and day 0 are shown. Scale bar: 100 µm. (i) Quantification of BrdU+ nuclei (n=3 independent experiments, each performed in duplicates or triplicates with 5 fields of view evaluated per individual replicate). #p<0.05 compared to respective condition at day -1. ∗p<0.05, ∗∗p<0.01, ∗∗∗p<0.001. **Supplementary Figure 2: *Picalm* Knockdown Alters Plasma Membrane Levels of Integrin Receptors but not Growth Hormone Receptors.** (a) Pathway enrichment analysis (gene ontology – biological process, GO-BP) using all plasma membrane differentially abundant proteins (DAPs) identified at day 2. Dot size represents the count of DAPs within each term and color indicates the percentage of upregulated DAPs per term. (b) Plasma membrane levels of integrin receptors and (c) growth hormone receptors at day 0, detected by proteome analysis. ∗p<0.05, ∗∗p<0.01 by students t-test with Welch correction. **Supplementary Figure 3: *Picalm* Depletion Does Not Alter Mitochondrial Capacity or Density.** (a-c) Mitochondrial capacity in *Picalm*-depleted C2C12 cells during differentiation (siRNA applied at day -2) was assessed by measuring oxygen consumption rates (OCR) using the Seahorse Extracellular Flux Analyzer in si*Picalm* and siNT cells under basal conditions and after subsequent injections of oligomycin, FCCP and rotenone combined with antimycin A. Values represent mean ± SEM (n=3 independent experiments, each performed with at least 10 replicates). (d) Mitochondrial capacity in mature C2C12 treated with *Picalm*-targeting siRNA after completion of differentiation process (siRNA applied day 4-6). (e) Basal respiration, ATP-linked respiration and maximal respiration and spare respiratory capacity calculated from OCR data in (a-d). (f) Mitochondrial DNA (mitoDNA) content was measured by qRT-PCR using genomic DNA as template. Mitochondrial DNA-encoded genes were normalized to an endogenous nuclear DNA control gene (Chdh1) to determine the relative abundance of mitochondrial DNA (n=2-4 independent experiments, each performed in duplicates to triplicates). (g) Experimental design for siRNA-mediated downregulation of *Picalm* in mature C2C12 myotubes after 4 days of differentiation, with experiments conducted on day 6, 48h after siRNA application. (h) Efficiency of *Picalm* downregulation on protein level in si*Picalm* cells compared to cells treated with non-targeting siRNA (siNT) on day 6 (n=3). (i) Western blot analysis of insulin-stimulated Akt phosphorylation in siNT and si*Picalm* C2C12 cells at day 6. Cells were serum- and nutrient-starved for 6h before stimulation with indicated insulin concentrations for 30 min. (j) Quantification of relative Akt phosphorylation levels at the indicated insulin concentrations (n=3). Multimedia component 1

## References

[1] Defronzo R.A., Jacot E., Jequier E., Maeder E., Wahren J., Felber J.P. (1981). The effect of insulin on the disposal of intravenous glucose: results from indirect calorimetry and hepatic and femoral venous catheterization. Diabetes.

[2] Richter E.A., Hargreaves M. (2013). Exercise, GLUT4, and skeletal muscle glucose uptake. Physiol Rev.

[3] Pedersen B.K., Febbraio M.A. (2012). Muscles, exercise and obesity: skeletal muscle as a secretory organ. Nat Rev Endocrinol.

[4] Prado C.M., Batsis J.A., Donini L.M., Gonzalez M.C., Siervo M. (2024). Sarcopenic obesity in older adults: a clinical overview. Nat Rev Endocrinol.

[5] Ando K., Nagaraj S., Küçükali F., de Fisenne M.A., Kosa A.C., Doeraene E. (2022). PICALM and Alzheimer's disease: an update and perspectives. Cells.

[6] Zhao Z., Sagare A.P., Ma Q., Halliday M.R., Kong P., Kisler K. (2015). Central role for PICALM in amyloid-β blood-brain barrier transcytosis and clearance. Nat Neurosci.

[7] Xiao Q., Gil S.C., Yan P., Wang Y., Han S., Gonzales E. (2012). Role of phosphatidylinositol clathrin assembly lymphoid-myeloid leukemia (PICALM) in intracellular amyloid precursor protein (APP) processing and amyloid plaque pathogenesis. J Biol Chem.

[8] Kanatsu K., Morohashi Y., Suzuki M., Kuroda H., Watanabe T., Tomita T. (2014). Decreased CALM expression reduces Aβ42 to total Aβ ratio through clathrin-mediated endocytosis of γ-secretase. Nat Commun.

[9] Moreau K., Fleming A., Imarisio S., Lopez Ramirez A., Mercer J.L., Jimenez-Sanchez M. (2014). PICALM modulates autophagy activity and tau accumulation. Nat Commun.

[10] Maritzen T., Koo S.J., Haucke V. (2012). Turning CALM into excitement: AP180 and CALM in endocytosis and disease. Biol Cell.

[11] Moreau K., Renna M., Rubinsztein D.C. (2013). Connections between SNAREs and autophagy. Trends Biochem Sci.

[12] Ishikawa Y., Maeda M., Pasham M., Aguet F., Tacheva-Grigorova S.K., Masuda T. (2015). Role of the clathrin adaptor PICALM in normal hematopoiesis and polycythemia vera pathophysiology. Haematologica.

[13] Suzuki M., Tanaka H., Tanimura A., Tanabe K., Oe N., Rai S. (2012). The clathrin assembly protein PICALM is required for erythroid maturation and transferrin internalization in mice. PLoS One.

[14] Huang F., Khvorova A., Marshall W., Sorkin A. (2004). Analysis of clathrin-mediated endocytosis of epidermal growth factor receptor by RNA interference. J Biol Chem.

[15] Harel A., Wu F., Mattson M.P., Morris C.M., Yao P.J. (2008). Evidence for CALM in directing VAMP2 trafficking. Traffic.

[16] Gaugel J., Haacke N., Sehgal R., Jähnert M., Jonas W., Hoffmann A. (2024). Picalm, a novel regulator of GLUT4-trafficking in adipose tissue. Mol Metabol.

[17] Baumeier C., Kaiser D., Heeren J., Scheja L., John C., Weise C. (2015). Caloric restriction and intermittent fasting alter hepatic lipid droplet proteome and diacylglycerol species and prevent diabetes in NZO mice. Biochim Biophys Acta Mol Cell Biol Lipids.

[18] Quiclet C., Dittberner N., Gässler A., Stadion M., Gerst F., Helms A. (2019). Pancreatic adipocytes mediate hypersecretion of insulin in diabetes-susceptible mice. Metabolism.

[19] Kanzleiter T., Jähnert M., Schulze G., Selbig J., Hallahan N., Schwenk R.W. (2015). Exercise training alters DNA methylation patterns in genes related to muscle growth and differentiation in mice. Am J Physiol Endocrinol Metab.

[20] Erickson M.L., Zhang H.U.I., Mey J.T., Kirwan J.P. (2020). Exercise training impacts skeletal muscle clock machinery in prediabetes. Med Sci Sports Exerc.

[21] Raza G.S., Kaya Y., Stenbäck V., Sharma R., Sodum N., Mutt S.J. (2024). Effect of aerobic exercise and time-restricted feeding on metabolic markers and circadian rhythm in mice fed with the high-fat diet. Mol Nutr Food Res.

[22] Zhao L., Hutchison A.T., Wittert G.A., Thompson C.H., Lange K., Liu B. (2020). Intermittent fasting does not uniformly impact genes involved in circadian regulation in women with obesity. Obesity.

[23] De Marinis Y., Sun J., Bompada P., Domènech Omella J., Luan C., Halu A. (2017). Regulation of nuclear receptor interacting protein 1 (NRIP1) gene expression in response to weight loss and exercise in humans. Obesity.

[24] Hoffmann C., Schneeweiss P., Randrianarisoa E., Schnauder G., Kappler L., Machann J. (2020). Response of mitochondrial respiration in adipose tissue and muscle to 8 weeks of endurance exercise in obese subjects. J Clin Endocrinol Metab.

[25] Chal J., Pourquié O. (2017). Making muscle: skeletal myogenesis in vivo and in vitro. Devenir.

[26] Miller S.E., Sahlender D.A., Graham S.C., Höning S., Robinson M.S., Peden A.A. (2011). The molecular basis for the endocytosis of small R-SNAREs by the clathrin adaptor CALM. Cell.

[27] Fader C.M., Sánchez D.G., Mestre M.B., Colombo M.I. (2009). TI-VAMP/VAMP7 and VAMP3/cellubrevin: two v-SNARE proteins involved in specific steps of the autophagy/multivesicular body pathways. Biochim Biophys Acta Mol Cell Res.

[28] Wu Y.T., Tan H.L., Shui G., Bauvy C., Huang Q., Wenk M.R. (2010). Dual role of 3-methyladenine in modulation of autophagy via different temporal patterns of inhibition on class I and III phosphoinositide 3-kinase. J Biol Chem.

[29] Watanabe N., Kato T., Fujita A., Ishizaki T., Narumiya S. (1999). Cooperation between mDia1 and ROCK in rho-induced actin reorganization. Nat Cell Biol.

[30] Zinn A., Goicoechea S.M., Kreider-Letterman G., Maity D., Awadia S., Cedeno-Rosario L. (2019). The small GTPase RhoG regulates microtubule-mediated focal adhesion disassembly. Sci Rep.

[31] Duan R., Gallagher P.J. (2009). Dependence of myoblast fusion on a cortical actin wall and nonmuscle myosin IIA. Dev Biol.

[32] Millay D.P., Rourke J.R.O., Sutherland L.B., Bezprozvannaya S., Shelton J.M., Bassel-duby R. (2013). Myomaker is a membrane activator of myoblast fusion and muscle formation. Nature.

[33] Zhang Q., Vashisht A.A., O'Rourke J., Corbel S.Y., Moran R., Romero A. (2017). The microprotein minion controls cell fusion and muscle formation. Nat Commun.

[34] Quinn M.E., Goh Q., Kurosaka M., Gamage D.G., Petrany M.J., Prasad V. (2017). Myomerger induces fusion of Non-fusogenic cells and is required for skeletal muscle development. Nat Commun.

[35] Bi P., Ramirez-martinez A., Li H., Cannavino J., Mcanally J.R., Shelton J.M. (2017). Control of muscle formation by the fusogenic micropeptide myomixer. Science.

[36] Gamage D.G., Melikov K., Munoz-Tello P., Wherley T.J., Focke L.C., Leikina E. (2022). Phosphatidylserine orchestrates Myomerger membrane insertions to drive myoblast fusion. Proc Natl Acad Sci USA.

[37] Sutton E.F., Beyl R., Early K.S., Cefalu W.T., Ravussin E., Peterson C.M. (2018). Early time-restricted feeding improves insulin sensitivity, blood pressure, and oxidative stress Even without weight loss in men with prediabetes. Cell Metab.

[38] Wilkinson M.J., Manoogian E.N.C., Zadourian A., Lo H., Fakhouri S., Shoghi A. (2020). Ten-hour time-restricted eating reduces weight, blood pressure, and atherogenic lipids in patients with metabolic syndrome. Cell Metab.

[39] Di Francesco A., Di Germanio C., Bernier M., De Cabo R. (2018). A time to fast. Science.

[40] Kleinert M., Parker B.L., Jensen T.E., Raun S.H., Pham P., Han X. (2018). Quantitative proteomic characterization of cellular pathways associated with altered insulin sensitivity in skeletal muscle following high-fat diet feeding and exercise training. Sci Rep.

[41] Pillon N.J., Gabriel B.M., Dollet L., Smith J.A.B., Sardón Puig L., Botella J. (2020). Transcriptomic profiling of skeletal muscle adaptations to exercise and inactivity. Nat Commun.

[42] Chen W., Xu Z., You W., Zhou Y., Wang L., Huang Y. (2023). Cold exposure alters lipid metabolism of skeletal muscle through HIF-1α-induced mitophagy. BMC Biol.

[43] Scripture-Adams D.D., Chesmore K.N., Barthélémy F., Wang R.T., Nieves-Rodriguez S., Wang D.W. (2022). Single nuclei transcriptomics of muscle reveals intra-muscular cell dynamics linked to dystrophin loss and rescue. Commun Biol.

[44] Meyerholz A., Hinrichsen L., Groos S., Esk P.C., Brandes G., Ungewickell E.J. (2005). Effect of clathrin assembly lymphoid myeloid leukemia protein depletion on clathrin coat formation. Traffic.

[45] Miller S.E., Mathiasen S., Bright N.A., Pierre F., Kelly B.T., Kladt N. (2015). CALM regulates clathrin-coated vesicle size and maturation by directly sensing and driving membrane curvature. Dev Cell.

[46] Takai Y., Sasaki T., Matozaki T. (2001). Small GTP-binding proteins. Physiol Rev.

[47] Klooster J.P., Hordijk P.L. (2007). Targeting and localized signalling by small GTPases. Biol Cell.

[48] Lee S.J., Seo B.R., Koh J.Y. (2015). Metallothionein-3 modulates the amyloid β endocytosis of astrocytes through its effects on actin polymerization. Mol Brain.

[49] Altankov G., Grinnell F. (1993). Depletion of intracellular potassium disrupts coated pits and reversibly inhibits cell polarization during fibroblast spreading. J Cell Biol.

[50] Jones M.C., Caswell P.T., Norman J.C. (2006). Endocytic recycling pathways: emerging regulators of cell migration. Curr Opin Cell Biol.

[51] Vassilopoulos S., Gentil C., Lainé J., Buclez P.O., Franck A., Ferry A. (2014). Actin scaffolding by clathrin heavy chain is required for skeletal muscle sarcomere organization. J Cell Biol.

[52] Shin N.Y., Choi H., Neff L., Wu Y., Saito H., Ferguson S.M. (2014). Dynamin and endocytosis are required for the fusion of osteoclasts and myoblasts. J Cell Biol.

[53] Boppart M.D., Mahmassani Z.S. (2019). Integrin signaling: linking mechanical stimulation to skeletal muscle hypertrophy. Am J Physiol Cell Physiol.

[54] Martin-Bermudo M.D., Dunin-Borkowski O.M., Brown N.H. (1998). Modulation of integrin activity is vital for morphogenesis. J Cell Biol.

[55] Wilschut K.J., van Tol H.T.A., Arkesteijn G.J.A., Haagsman H.P., Roelen B.A.J. (2011). Alpha 6 integrin is important for myogenic stem cell differentiation. Stem Cell Res.

[56] Han J.W., Lee H.J., Bae G.U., Kang J.S. (2011). Promyogenic function of Integrin/FAK signaling is mediated by Cdo, Cdc42 and MyoD. Cell Signal.

[57] Hattersley K.J., Carosi J.M., Hein L.K., Bensalem J., Sargeant T.J. (2021). PICALM regulates cathepsin D processing and lysosomal function. Biochem Biophys Res Commun.

[58] Tajika Y., Takahashi M., Khairani A.F., Ueno H., Murakami T., Yorifuji H. (2014). Vesicular transport system in myotubes: ultrastructural study and signposting with vesicle-associated membrane proteins. Histochem Cell Biol.

[59] Takahashi M., Tajika Y., Khairani A.F., Ueno H., Murakami T., Yorifuji H. (2013). The localization of VAMP5 in skeletal and cardiac muscle. Histochem Cell Biol.

[60] Zeng Q., Subramaniam V.N., Wong S.H., Tang B.L., Parton R.G., Rea S. (1998). A novel synaptobrevin/VAMP homologous protein (VAMP5) is increased during in vitro myogenesis and present in the plasma membrane. Mol Biol Cell.

[61] Kovac L., Goj T., Ouni M., Irmler M., Jähnert M., Beckers J. (2024). Skeletal muscle gene expression signatures of Obese high and low responders to endurance exercise training. J Clin Endocrinol Metab.

[62] Gromova A., Tierney M., Sacco A. (2015). FACS-based satellite cell isolation from mouse hind limb muscles. Bio-protocol.

[63] Shahini A., Vydiam K., Choudhury D., Rajabian N., Nguyen T., Lei P. (2018). Efficient and high yield isolation of myoblasts from skeletal muscle. Stem Cell Res.

[64] Bolte S., Cordelières F.P. (2006).

[65] Tyanova S., Temu T., Cox J. (2016). The MaxQuant computational platform for mass spectrometry-based shotgun proteomics. Nat Protoc.

[66] Tyanova S., Temu T., Sinitcyn P., Carlson A., Hein M.Y., Geiger T. (2016). The Perseus computational platform for comprehensive analysis of (prote)omics data. Nat Methods.

