# Supplementary material for: Picalm coordinates clathrin-mediated endocytosis and actin remodeling during myogenesis
Source: Mol Metab. 2026 Mar 13;107:102351. doi: 10.1016/j.molmet.2026.102351 (PMC13049958; doi:10.1016/j.molmet.2026.102351)
Supplement: Multimedia component 1 — Supplementary Figure 1: Adaptations of the Plasma Membrane Proteome in Response to Picalm Knockdown. (a) Co-localization of Picalm with the endosomal marker EEA1 and the AP2 adaptor complex in C2C12 cells (day 2); nuclei were stained using DAPI. Scale bar: 10 µm. (b) Pearson‘s correlation coefficient between fluorescent signal of Picalm (red) and EEA1 or AP2 (green) per field of view. (c) Enrichment of the plasma membrane marker Na+/K+ ATPase in the plasma membrane-enriched fraction (PM) of C2C12 cells (day 0) retrieved by differential ultracentrifugation compared to high density microsomes (HDM) fraction and low density microsomes (LDM). (d) Subcellular localization of Vamp3 in siNT and siPicalm myoblasts: 48h after siRNA treatment, C2C12 myoblasts were fixated and stained using Vamp3- and Picalm-targeting antibodies, DAPI and Phalloidin to visualize f-actin. Scale bar: 10 µm. (e) Quantification of p62 by western blot analysis of cells treated with the autophagic modulator Chloroquine (CQ) performed on day 0 for 6h (n=3 independent experiments, performed in duplicates or dublicates). (f) Western blot analysis of cleaved caspase 3 levels in siPicalm and siNT C2C12 cells during differentiation, normalized to the housekeeping protein Gapdh. (g) Quantification of relative cleaved caspase 3 levels normalized to Gapdh, expressed relative to siNT levels (n=3 independent experiments, performed in duplicates or triplicates). (h) Proliferation capacity measured on day -1, day 0 and day 2 by BrdU incorporation assay. BrdU (50 µM) was applied to C2C12 cells for 2h before fixation of nuclei staining (DAPI). Representative images for day -1 and day 0 are shown. Scale bar: 100 µm. (i) Quantification of BrdU+ nuclei (n=3 independent experiments, each performed in duplicates or triplicates with 5 fields of view evaluated per individual replicate). #p<0.05 compared to respective condition at day -1. ∗p<0.05, ∗∗p<0.01, ∗∗∗p<0.001. Supplementary Figure 2: Picalm Knockdown Alters Plasm [file mmc1.pptx]

## Slide 1
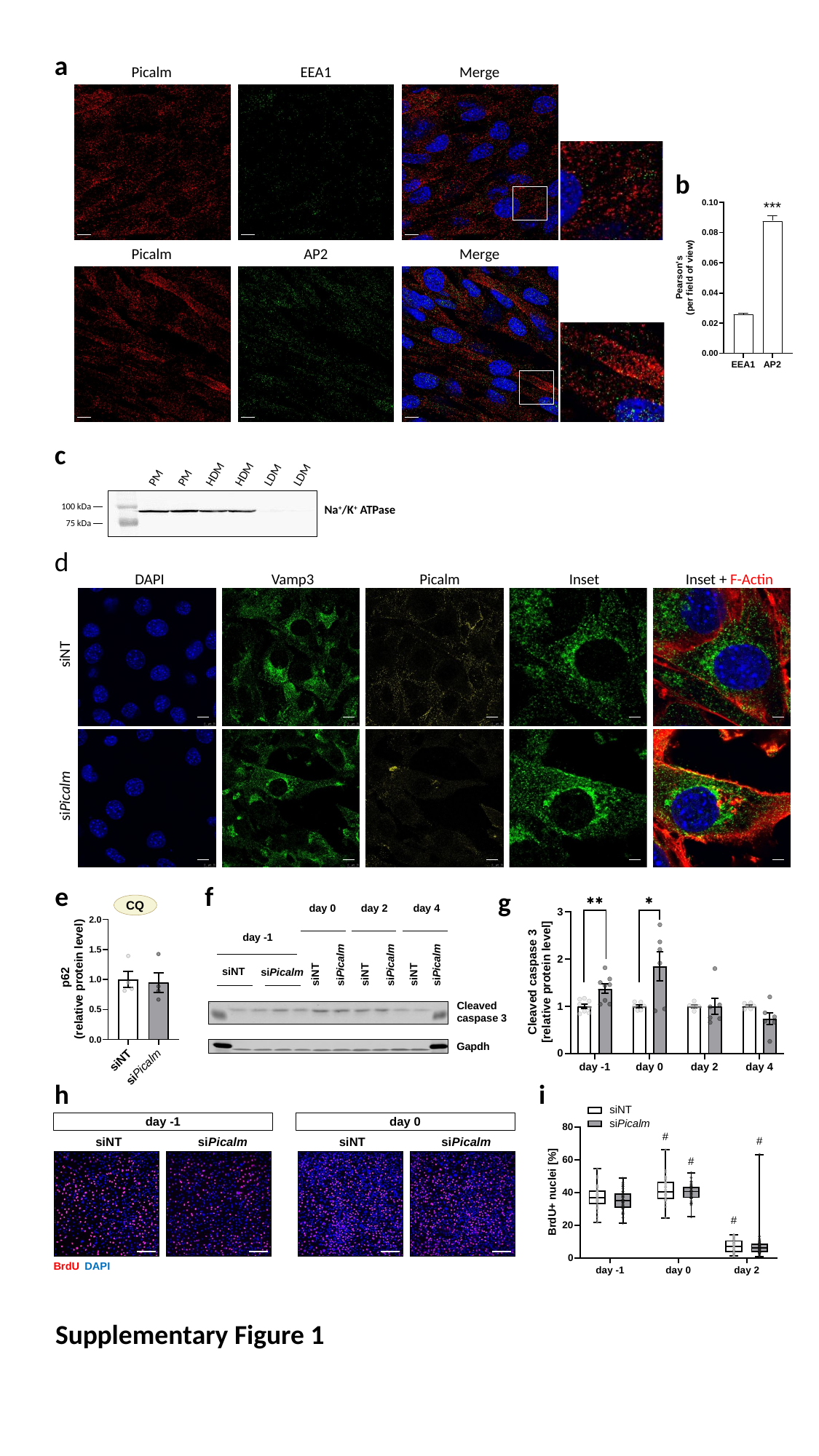

a
Picalm
EEA1
Merge
b
Picalm
AP2
Merge
c
HDM
HDM
LDM
LDM
PM
PM
100 kDa
Na+/K+ ATPase
75 kDa
d
DAPI
Vamp3
Picalm
Inset
Inset + F-Actin
siNT
siPicalm
e
f
g
CQ
day 2
day 4
day 0
day -1
siPicalm
siPicalm
siPicalm
siNT
siPicalm
siNT
siNT
siNT
Cleaved
caspase 3
Gapdh
h
i
day -1
day 0
siNT
siPicalm
siNT
siPicalm
i
BrdU
DAPI
Supplementary Figure 1

## Slide 2
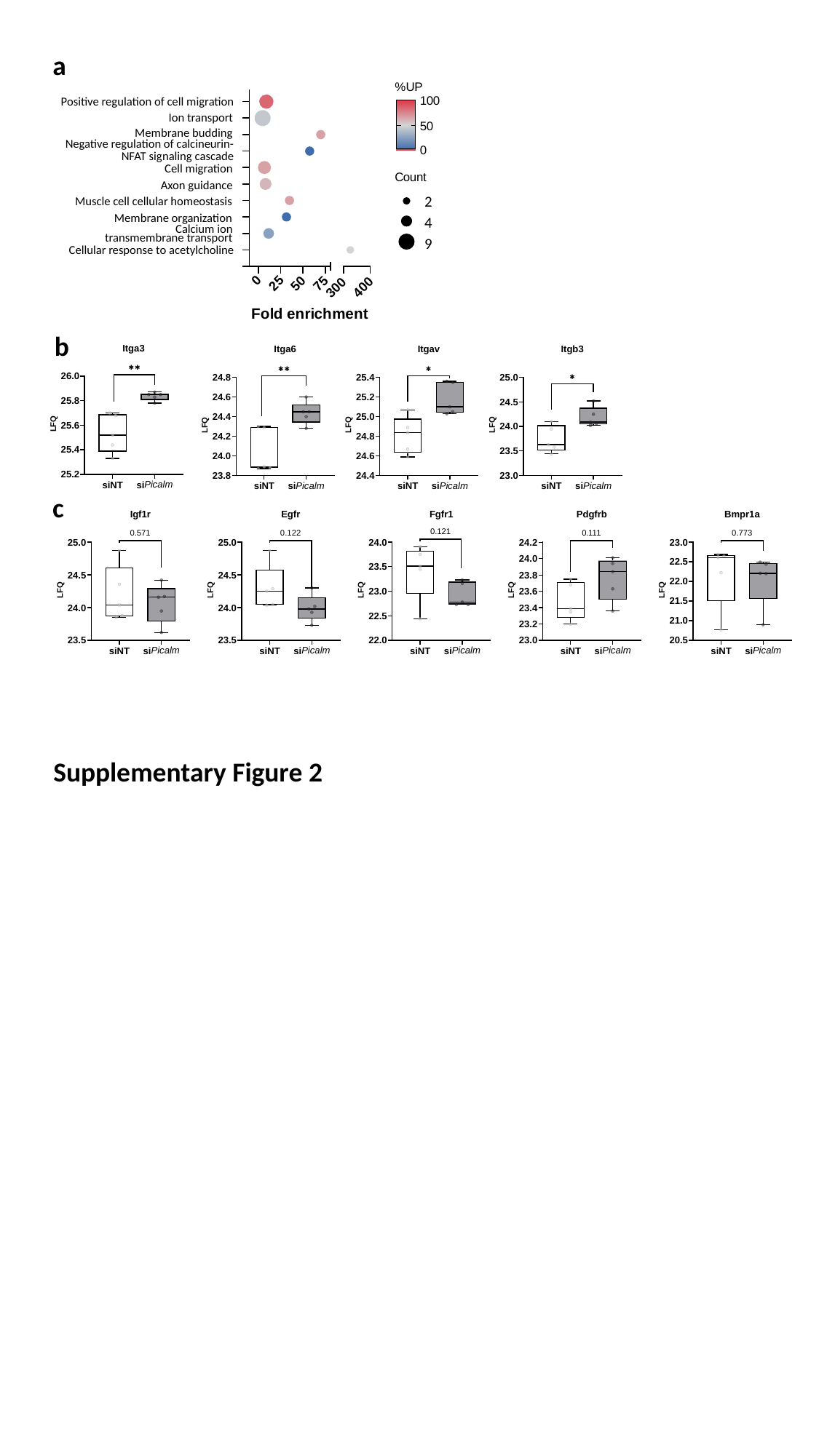

a
Positive regulation of cell migration
Ion transport
Membrane budding
Negative regulation of calcineurin-
NFAT signaling cascade
Cell migration
Axon guidance
Muscle cell cellular homeostasis
Membrane organization
Calcium ion
transmembrane transport
Cellular response to acetylcholine
2
4
9
b
c
Supplementary Figure 2

## Slide 3
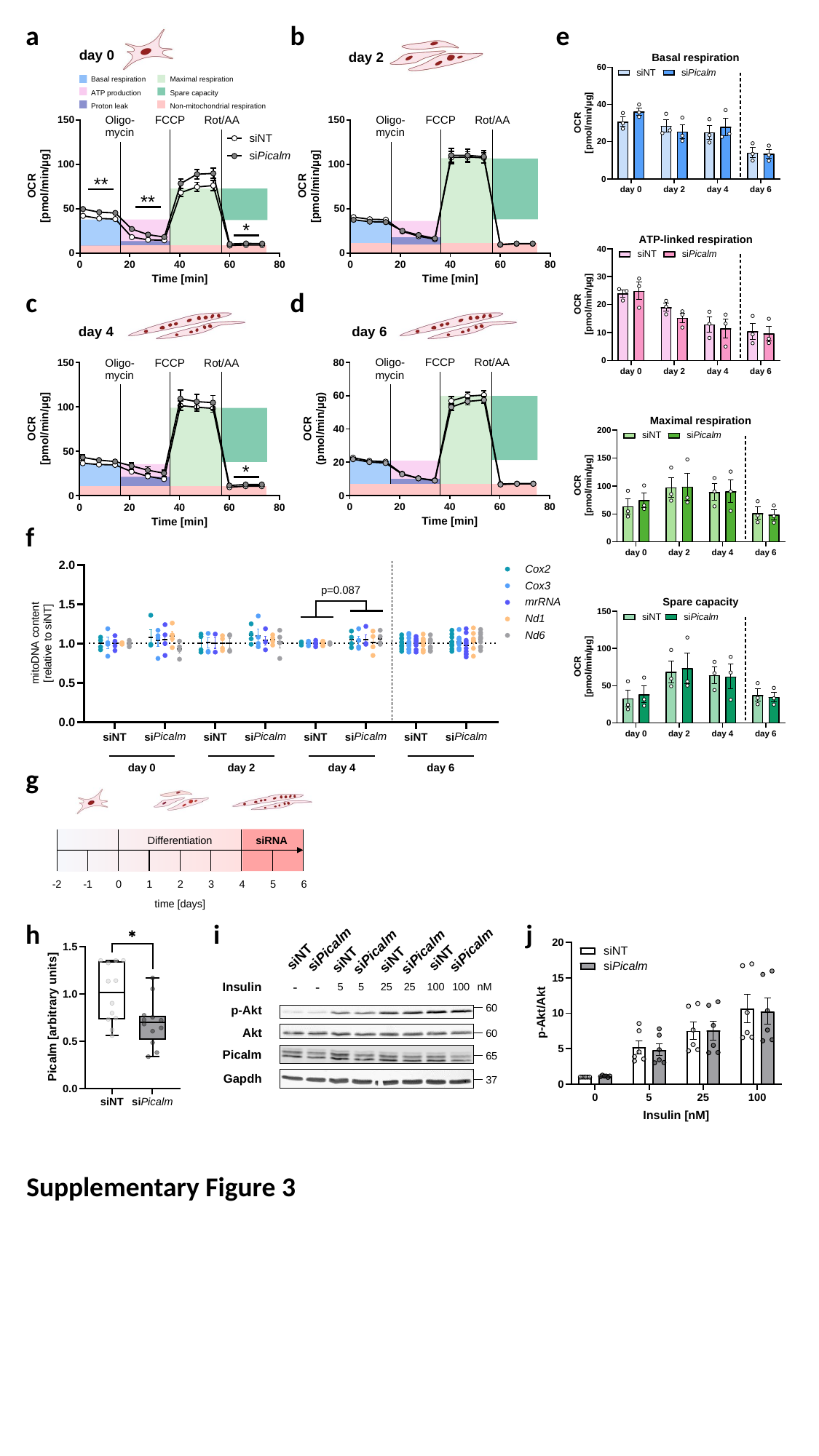

a
b
e
day 0
day 2
c
d
day 4
day 6
f
g
siRNA
Differentiation
-2
-1
0
1
2
3
4
5
6
time [days]
siPicalm
siPicalm
siPicalm
siPicalm
siNT
siNT
siNT
siNT
Insulin
5
5
25
25
100
100
p-Akt
Akt
Picalm
Gapdh
60
60
65
37
nM
h
i
j
Supplementary Figure 3
